# Identifying markers of health-seeking behaviour and healthcare access in UK electronic health records

**DOI:** 10.1136/bmjopen-2023-081781

**Published:** 2024-09-26

**Authors:** Sophie Graham, Jemma L Walker, Nick Andrews, Dorothea Nitsch, Edward P K Parker, Helen McDonald

**Affiliations:** 1 London School of Hygiene & Tropical Medicine Faculty of Epidemiology and Public Health, London, UK; 2 Faculty of Epidemiology and Population Health, London School of Hygiene and Tropical Medicine, London, UK; 3 UK Health Security Agency, London, UK; 4 UK Renal Registry, Bristol, UK; 5 Renal Unit, Royal Free London NHS Foundation Trust, Hertfordshire, UK; 6 University of Bath, Bath, UK

**Keywords:** primary health care, vaccination, epidemiologic studies, health services

## Abstract

**Abstract:**

**Objective:**

To assess the feasibility of identifying markers of health-seeking behaviour and healthcare access in UK electronic health records (EHR), for identifying populations at risk of poor health outcomes and adjusting for confounding in epidemiological studies.

**Design:**

Cross-sectional observational study using the Clinical Practice Research Datalink Aurum prelinked to Hospital Episode Statistics.

**Setting:**

Individual-level routine clinical data from 13 million patients across general practices (GPs) and secondary data in England.

**Participants:**

Individuals aged ≥66 years on 1 September 2019.

**Main outcome measures:**

We used the Theory of Planned Behaviour (TPB) model and the literature to iteratively develop criteria for markers selection. Based on this we selected 15 markers: those that represented uptake of public health interventions, markers of active healthcare access/use and markers of lack of access/underuse. We calculated the prevalence of each marker using relevant lookback periods prior to the index date (1 September 2019) and compared with national estimates. We assessed the correlation coefficients (phi) between markers with inferred hierarchical clustering.

**Results:**

We included 1 991 284 individuals (mean age: 75.9 and 54.0% women). The prevalence of markers ranged from <0.1% (low-value prescriptions) to 92.6% (GP visits), and most were in line with national estimates; for example, 73.3% for influenza vaccination in the 2018/2019 season, compared with 72.4% in national estimates. Screening markers, for example, abdominal aortic aneurysm screening were under-recorded even in age-eligible groups (54.3% in 65–69 years old vs 76.1% in national estimates in men). Overall, marker correlations were low (<0.5) and clustered into groups according to underlying determinants from the TPB model.

**Conclusion:**

Overall, markers of health-seeking behaviour and healthcare access can be identified in UK EHRs. The generally low correlations between different markers of health-seeking behaviour and healthcare access suggest a range of variables are needed to capture different determinants of healthcare use.

**Strengths and limitations of this study**

- This is the first known study in the UK that has identified proxies or markers of health-seeking behaviour or healthcare access.
- We used linked electronic health records from primary and secondary care so that a range of different health utilisation markers could be identified.
- We identified a large population of over 2 million individuals.
- For some of the markers (eg, bone density scans), health need could not be entirely separated from health behaviour and access.
- Marker prevalences showed different patterns by age, and these findings might not be generalisable to younger age groups (<65 years).

## Background

 Health-seeking behaviour can be defined as ‘any activity undertaken by a person believing [themselves] to be healthy, for the purpose of preventing disease or detecting it in an asymptomatic stage’.^1^ Healthcare access can be defined as ‘the ability to obtain healthcare services such as prevention, diagnosis, treatment, and management of diseases, illness, disorders, and other health-impacting conditions’.^2^ Healthcare professionals or researchers might be interested in identifying patients with a lack of health-seeking behaviour or healthcare access, since these individuals are likely to suffer from worse clinical outcomes. Health-seeking behaviour and healthcare access may also be a key confounder in observational studies, and failure to account for this may undermine the validity of results. This type of confounding is thought to have contributed to overestimates of the protective effect of influenza vaccinations against all-cause mortality in observational cohort studies.^3^ Information on health-seeking behaviour and healthcare access can be collected prospectively through surveys or interviews; for example, in the English Longitudinal Study of Ageing study.^4^ Typically, in routinely-recorded data such as electronic health records (EHRs) it is difficult to identify health-seeking behaviour and healthcare access since they are not directly recorded. Suitable markers would need to represent interactions with the healthcare system (ie, healthcare utilisation), preferably with limited dependence on underlying health needs. Behavioural scientists have a variety of models for explaining the determinants for healthcare utilisation. For example, the updated Theory of Planned Behaviour (TPB) model^5^ describes the psychological, physical, contextual and socio-demographic determinants for healthcare utilisation. Psychological determinants include influences on the micro and macro level such as societal attitudes, but also personal prior experiences. Physical determinants are on the micro-level and include lifestyle factors such as drug consumption, body mass index and physical activity. Context determinants are on the macro-level and include potential external barriers such as recommendations from healthcare professionals or geopolitical influences. Socio-demographic determinants are on the micro-level and include individual characteristics such as sex, age and living arrangements. These models demonstrate that there are a range of different determinants and therefore many different markers are likely required to capture all the underlying influences.

Three recent studies in the USA^6^,^8^ introduced adjusting for markers of health-seeking behaviour and healthcare access in observational research. However, it is not known to what extent suitable markers can be identified in UK EHR. This study aimed to identify markers of health-seeking behaviour and healthcare access in UK EHRs, compare their prevalence to available national estimates and explore correlations between different markers. This study will focus on individuals aged over 65 years as health-seeking behaviour and healthcare access vary by age^9^ and because they have high morbidity and mortality.^10^

## Methods

### Data sources, study design and population

We used the Clinical Practice Research Datalink (CPRD) Aurum prelinked to Hospital Episode Statistics (HES) admitted patient care (APC). For more information on this data source, see table 1.

**Table 1:** Description of the CPRD Aurum-HES APC data sources

	CPRD-HES
Type of data set	CPRD Aurum includes anonymised longitudinal primary care patient records collected from the EMIS Health patient record system. HES APC is a secondary care commissioning data set that covers all NHS secondary care in England.^26^
Population coverage	At the time of data extraction (CPRD Aurum May 2022 release) this data included 1491 currently contributing general practices for 13 300 067 currently contributing patients (19.83% of the UK population). Only patients with linkage to HES can be identified via CPRD Aurum.
Geographical coverage	99% of the GP are in England and <1% are in Northern Ireland.^27^
Coding systems used	CPRD Aurum uses a combination of SNOMED, Read codes (Clinical Terms V.3) and local EMIS codes that are each individually mapped to a unique ‘medcode’. Prescriptions are recorded using the NHS dictionary of medicines and devices, each are mapped to a unique prescription code. HES uses International Classification of Diseases 10th revision codes 13 to record diagnoses and Classification of Interventions and Procedures (OPCS) codes 14 to record procedures.
Representativeness	The data are representative of the broader English population in terms of age, gender, geographical spread and deprivation as compared with Office for National Statistics.^28^

APC, admitted patient care; CPRD, Clinical Practice Research Datalink; GPgeneral practiceHES, Hospital Episode Statistics; NHSNational Health ServiceOPCSOffice of Population Censuses and SurveysSNOMED, Systematised Nomenclature of Medicine Clinical Terms

This was a cross-sectional study design that included a study population of individuals in England aged 66 years or older on 1 September 2019 identified in the CPRD-HES data. 1 September 2019 was selected as the index date for all individuals. We only included individuals with a general practice (GP) registration start date before 1 September 2018 to allow for a minimum 1-year pre-index period for marker identification.

### Marker selection

We used the TPB model to define our aim of identifying healthcare utilisation driven by determinants other than physical and mental health. We selected candidate markers available within the CPRD Aurum-HES data using our own formal criteria (see table 2). The criteria were developed with input from two clinical epidemiologists on UK clinical practice and data recording (DN and HIM). Candidate markers from the aforementioned US studies^6^,^8^ were tested against our developed criteria to iteratively make improvements to the criteria and to identify additional potential markers. Candidate markers that failed to meet our prespecified criteria included interactions linked with underlying health needs (eg, for cardiovascular disease screening, depression screening, hospital visits) and programmes not available to all individuals (eg, Shingrix vaccination; see online supplemental table 1 for a full list of excluded markers). We selected 15 markers that included abdominal aortic aneurysm (AAA) screening; breast cancer screening; bowel cancer screening; cervical cancer screening; influenza vaccination; pneumococcal vaccination; National Health Service (NHS) health checks; prostate-specific antigen (PSA) testing; bone density scans; low-value procedures; glucosamine use (low-value prescription); GP visits; did not attend (DNA) primary care visit; hospital visit for ambulatory care sensitive (ACS) condition; and blood pressure measurements. In general, the criteria were a good fit for the markers, but there was some tolerance for minor deviations, particularly for accepting some influence of underlying health conditions (online supplemental table 2).

**Table 2:** Criteria used to assess inclusion of markers of health-seeking behaviour and healthcare access

#	Criteria	Explanation	Example of a marker that does not meet criteria
1	Should be currently or recently available in national clinical practice to all individuals (overall or by sex) at cohort entry.	Ensures that the denominator population (by sex) is eligible for each of the markers.	Shingles vaccination is currently recommended in the UK to all individuals turning 65 years (among others).^1^ However, it was not historically available for all age-cohorts in this study due to the evolving age-based eligibility criteria since vaccine introduction in 2013. As a result, only selected age-cohorts would have had a period of age-based eligibility for shingles vaccination at the study index date, and this would not have been a universal marker for the study population.
2	Should be routinely recorded in the available data sources.	Ensures routine ascertainment of markers which is not dependent on other factors such as abnormal test results.	Vision and hearing tests are available through the NHS in the UK; however, most people get these tests from a private optician. Although opticians routinely send results to GPs, these may be uploaded as a PDF rather than coded in the patient’s health record, particularly if no abnormality is found.
3	Should not be strongly dependent on underlying health needs.	Ensures that the determinants of healthcare utilisation are not strongly driven by underlying health conditions.	Adherence to medication could represent health-seeking behaviour and healthcare access; however, medication use is dependent on a diagnosed condition or health need.

Note: Shingles vaccine was first made available to immunocompetent individuals aged 70 or 79 in 2013 in the UK, with a phased catch-up programme for individuals aged 70–79 years. In 2021, the programme introduced recombinant vaccination which can be given to people with immunosuppression. At the time of the study index date, shingles vaccine was available to all individuals aged 70–79 years. Shingles vaccination is currently (1 September 2023) recommended in the UK to all individuals turning 65 years, currently aged 70–79, or aged 50 and over with immunosuppression.

GPgeneral practiceNHSNational Health Service

Some markers represented active health-seeking behaviour and healthcare access, such as the uptake of recommended vaccinations. Other markers represented a lack of health-seeking behaviour and healthcare access—such as DNA for primary care visits, and hospital visits for ACS conditions. ACS conditions are conditions for which effective community care can help prevent the need for hospital admission.^11^ If an individual has a visit to the hospital for an ACS condition, then this could signify either barriers to healthcare access on the macro level (eg, inundated GP), barriers on the individual level (eg, language barriers) or a lack of health-seeking behaviour. Low-value procedures and low-value prescriptions are those that the National Institutes of Health and Care Excellence recommended to no longer provide in UK clinical practice since they were deemed to have little or no benefit, while still incurring an avoidable cost.^12 13^ We considered both to be indicators of active health-seeking behaviour or healthcare access from a patient perspective, as patients were receiving (non-recommended) care for their perceived needs.

### Marker operational definitions

The operational definition of each marker includes code lists (sets of diagnostic or prescription codes that represent a given clinical concept^14^) and lookback periods to apply in the current study data sets (table 3).

**Table 3:** Use of markers in UK clinical practice and operational definitions

Marker	Use of this marker in current UK clinical practice	Operational definition	Sensitivity analysis	CPRD Aurum		HES APC	
				Medcode	Prescription code	ICD-10	OPCS
AAA screen	Available once to men when they turn 65 years.^29^	≥1 AAA screen identified ever before index.	Alternatively using a broad code list.*	✓			
Breast cancer screen	Available every 3 years to women aged 50–71 years.^18^	≥1 breast cancer screen identified from the last 4 years that they were age-eligible for screening until index date.	Alternatively using a restrictive lookback† and a broad code list.*	✓			
Cervical cancer screen	Available to women every 3 years between the ages of 25 and 49 years and every 5 years between the ages of 50 and 64 years.^30^	≥1 cervical cancer screen identified from the last 6 years that they were age-eligible for screening until index date.	Alternatively using a restrictive lookback.†	✓			
Bowel cancer screen	Available every 2 years to all individuals aged 60–74 years.^16^	≥1 bowel cancer screen identified from the last 3 years that they were age-eligible for screening until index date.	Alternatively using a restrictive lookback.†	✓			
NHS health checks	Available every 5 years to all individuals aged 40–74 years without pre-existing conditions.‡^31^	≥1 NHS health check identified from the last 6 years that they were age-eligible for NHS health checks until index date.	Alternatively using a restrictive lookback.†	✓			
Influenza vaccination	Available annually to all individuals during the influenza season (1 September to 31 March) to all individuals aged ≥65 years.^23^	≥1 influenza vaccination identified from 1 September 2018 to 31 March 2019. See online supplemental table 3 for vaccination algorithm using both medcodes and prescription codes.	None.	✓	✓		
Pneumococcal vaccination	Available once to all individuals when they turn 65 years, or earlier for those with pre-existing conditions‡.^32^	≥1 pneumococcal vaccination identified ever before index.	None.	✓	✓		
PSA test	Available to all men.^33^	≥1 PSA test identified in the 3 years before index.	None.	✓			
Bone density scans	Available to all individuals.^34^	≥1 bone density scan identified in the 3 years before index.	None.	✓			
GP visits	Available to all individuals.^35^	1 GP visit(s) identified in the 1 year before index identified using^36^ EMIS consultation source identifiers, consultation source code identifiers and job categories to identify GP and nurse visits (excluding out-of-hours visits).^36^	None.				
DNA primary care visit	Available to all individuals.^35^	≥1 DNA primary care visits identified in the 1 year before index.	None.	✓			
Low-value procedures	Available to all individuals.^12^	≥1 low-value procedures identified in the 1 year before index.	None.				✓
Low-value prescription (glucosamine)	Available to all individuals.^13^	≥1 low-value prescriptions identified in the 1 year before index.	None.		✓		
Hospital visit for ACS condition	Available to all individuals.^37^	≥1 hospital visits for an ACS condition identified in the 5 years before index.	None.			✓ (primary position only)	
Blood pressure measurements	Available to all individuals.^38^	≥1 blood pressure measurement identified in the 1 year before index.	None.	✓			

* Broad code lists: for screening markers where the diagnostic test can be used for symptoms, broad code lists would include the diagnostic test, but did not require ‘screen’ or ‘screening’ to be in the medcode.

† Restricted lookback: for markers with an upper eligible age, the lookback period would be stopped at the upper age of eligibility.

‡ Pre-existing conditions: chronic heart disease, chronic kidney disease, diabetes, high blood pressure, atrial fibrillation, transient ischaemic attack, inherited high cholesterol, heart failure, peripheral arterial disease, stroke, currently prescribed statins to lower cholesterol and previous checks that have found a 20% higher risk of getting cardiovascular disease over the next 10 years.^24^.

ACSambulatory care sensitiveCPRD, Clinical Practice Research Datalink; DNA, did not attend; GP, general practice; HES, Hospital Episode Statistics; ICD-10, International Classification of Diseases 10th revision; NHS, National Health Service; OPCS, Office of Population Censuses and Surveys; PSA, prostate-specific antigen

For code lists, existing validated code lists were used where possible. Primarily we searched for code lists that were incentivised for national use through the Quality and Outcomes Framework^15^ or those that were validated through research. If code lists were not available using these sources, then they were developed using keyword searches (based on Medical Subject Headings terms with corresponding synonyms). Where possible the code lists aimed to be as specific as possible (‘narrow code lists’) and therefore codes were excluded if they were not clearly relevant. For example, for most screening markers we required the code to specify ‘screen’ or ‘screening’ but for bowel cancer, we also allowed Faecal Immunochemical Tests (FIT) as these were in routine use in the national screening programme during the study period, but were not a first choice for symptomatic testing at the time.^16 17^ As a sensitivity analysis, for AAA, breast cancer and cervical cancer screening, since the same procedure may be recorded for a screening test as for diagnostic tests investigating symptoms, we also included a broader code list that included codes that specified the relevant procedure, but did not specify ‘screen’ or ‘screening’. Full inclusion and exclusion list were reviewed by a clinical epidemiologist (HIM) and differences were agreed upon discussion and third-party review (EPKP). The search terms that were used to create the code lists and the code lists that were used can be found on the LSHTM data compass (https://doi.org/10.17037/DATA.00003684).

The lookback periods for each marker were developed by first identifying how each of these markers are recommended for use in current UK clinical practice. For markers that are available to all at any time, the lookback period reflected the expected frequency of healthcare use in UK clinical practice. For example, for markers that were expected to be frequently recorded (eg, blood pressure measurements) we used a 1-year lookback. For markers that were expected to be less frequently recorded (eg, hospital visit for ACS conditions) a 5-year lookback was used. For markers with an upper age limit of eligibility (ie, screening and NHS health checks), we ensured the lookback period reflected the timely administration of these markers (since we were interested in capturing strong evidence of health-seeking behaviour and healthcare access). For example, breast cancer screening is offered to women every 3 years aged 50–71 years^18^ and therefore the lookback period covered the last 4 years of age-eligibility (3 years plus an additional year for the uncertainty of age as the only year of birth is recorded in CPRD) for breast cancer screening, until the index date. We included all follow-up time until the index date to allow for delayed recording due to the transition to electronic records among older individuals. As a sensitivity analysis, since we were concerned that including years after the upper age of eligibility might have meant we included more symptomatic individuals rather than healthy individuals accessing screening programmes, we also employed a restricted lookback that stopped the look back at the upper age of eligibility (see online supplemental figure 1).

### Prevalence estimates

For prevalence calculations, the denominator was all individuals aged ≥66 years on 1 September 2019 and the numerator was ≥1 occurrence of the marker in the relevant lookback period. We also calculated prevalence stratified by sex (given the inclusion of several sex-specific markers) and age in 5-year bands (65–69, 70–74, 75–79, 80–84, 85–89, 90–95 and 95+ years).

We compared prevalence estimates to national estimates from PHE fingertips or from published literature, preferentially selecting for recent estimates from the UK in the relevant age group. The prevalence estimates from these sources can be found in table 4 and sources are detailed in online supplemental table 4.

**Table 4:** Prevalence of markers

Variable	All individuals	Male	Female	National estimates	Theoretical grouping from TPB model*
N	1 991 284	915 561	1 075 723		
AAA screen†	231 088 (11.6%)	227 844 (24.9%)	3244 (0.3%)	76.1%	Contextual
Breast cancer screen†	346 116 (17.4%)	517 (0.1%)	345 599 (32.1%)	71.1%	Contextual
Cervical cancer screen†	397 303 (20.0%)	153 (0.0%)	397 150 (36.9%)	76.2%	Contextual
Bowel cancer screen†	1 439 412 (72.3%)	687 712 (75.1%)	751 700 (69.9%)	60.5%	Contextual
NHS health checks†‡	372 244 (18.7%)	157 484 (17.2%)	214 760 (20.0%)	40%§	Contextual
Influenza vaccine	1 460 391 (73.3%)	670 162 (73.2%)	790 229 (73.5%)	72.4%	Psychological
Pneumococcal vaccination	1 242 359 (62.4%)	568 798 (62.1%)	673 561 (62.6%)	69.0%	Psychological
PSA testing	352 272 (17.7%)	351 884 (38.4%)	388 (0.0%)	53.0%	Physical with active access
Bone density scan	100 892 (5.1%)	19 407 (2.1%)	81 485 (7.6%)	0.03–1.6%	Physical with active access
GP visits	1 844 823 (92.6%)	841 413 (91.9%)	1 003 410 (93.3%)	¶	Physical with active access
DNA primary care visit	601 896 (30.2%)	275 449 (30.1%)	326 447 (30.3%)	¶	Physical with lack of access
Low-value procedures	358 881 (18.0%)	168 746 (18.4%)	190 135 (17.7%)	0.02–0.2%	Physical with active access
Low-value prescription (glucosamine)	219 (0.0%)	75 (0.0%)	144 (0.0%)	¶	Physical with active access
Hospital visit for an ACS condition	190 136 (9.5%)	82 691 (9.0%)	107 445 (10.0%)	3–15%**	Physical with lack of access
Blood pressure measurement	1 470 006 (73.8%)	681 294 (74.4%)	788 712 (73.3%)	84.6%	Physical with active access

* Theoretical grouping from the updated Theory of Planned Behaviour model.^5^

**Figure 1:**
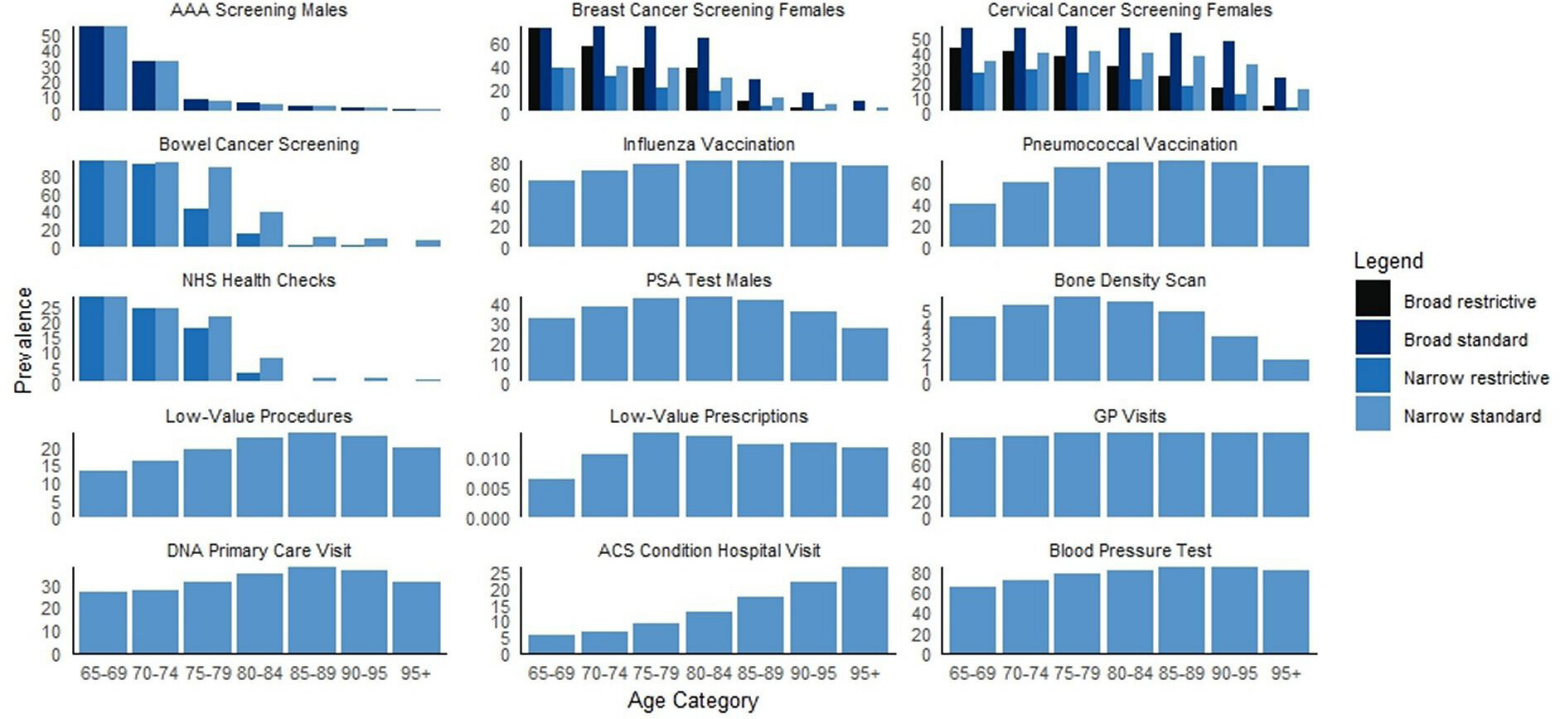
Prevalence of markers, stratified by age category. Note: the numbers and proportions for these bar charts can be found in online supplemental table 7. AAA, abdominal aortic aneurysm; ACS, ambulatory care sensitive; DNA, do not attend; GP, general practice; NHS, National Health Service; PSA, prostate-specific antigen.

† The denominator for the current study does not restrict to those that are age-eligible unlike in the national estimate. For age-eligible estimates see figure 1.

‡ The denominator for the current study does not exclude individuals without pre-existing conditions (chronic heart disease, chronic kidney disease, diabetes, high blood pressure, atrial fibrillation, transient ischaemic attack, inherited high cholesterol, heart failure, peripheral arterial disease, stroke, currently prescribed statins to lower cholesterol and previous checks that have found a 20% higher risk of getting cardiovascular disease over the next 10 years^31^) unlike in the national estimate.

§ The national estimate for NHS health checks is the percentage of eligible individuals receiving an NHS health check in Q1 2019/2020: 2.0%. To better match the lookback period for this marker (5-years years), we multiplied this estimate by 20.

¶ Prevalence of these markers are not knowingly presented in national estimates. NHS digital and OpenPrescribing report the total unit counts for these markers, which are reported in Supplementary Table 4online supplemental table 4.

** Estimate varies according to age strata.

AAA, abdominal aortic aneurysm; ACS, ambulatory care sensitive; DNA, did not attend; GP, general practice; NHSNational Health ServicePSA, prostate-specific antigen; TPB, Theory of Planned Behaviour

### Correlations

The correlation of all the markers within the population sample was assessed using a phi correlation matrix. The phi coefficient is designed to measure the association between binary variables, and is equivalent to a Pearson correlation when applied to binary data. It ranges from −1 to 1, where 0 signifies no relationship between the variables, 1 is a perfect positive relationship and −1 is a perfect negative relationship.^19^ Variables were ordered via complete linkage hierarchical clustering which was visualised by adorning dendrograms onto the correlation matrices (heatmaply_cor in R).

The clustering of markers was compared with a theoretical grouping using the updated TPB model.^5^ The theoretical grouping was based on the underlying determinants from the updated TPB model (table 4). Specifically, we grouped markers into four groups: those with strong psychological influences (‘psychologically determined’; eg, vaccinations), those with strong contextual influences (‘contextually determined’; eg, screening and NHS health checks) and those fully or partially dependent on physical need. Physically determined markers were further separated into those likely to represent lack of health-seeking behaviour or healthcare access (eg, DNA primary care visit and ACS condition hospital visit; ‘physically determined with lack of access’) and those likely to represent active health-seeking behaviour or straightforward healthcare access (‘physically determined with active access’).

All programming was conducted using R (V.4.2.1–4.2.3) and the programming code can be found on GitHub (https://github.com/grahams99/Health-seeking-behaviour).

### Patient and public involvement

No patient or public involvement in the study as anonymised patient data set was used in the analysis.

## Results

Overall, 1 991 284 individuals were included (54.0% women, mean (SD) age: 75.9 (7.4); online supplemental table 5).

The prevalence of markers in the overall population ranged from <0.1% for low-value prescriptions to 92.6% for GP visits. The proportion with at least one GP visit was so high that we conducted a post-hoc analysis that revealed the median (IQR) number of GP visits was 7 (4–11) with some patients having over 25 visits per year (online supplemental figure 2). The prevalence of markers was similar between men and women, except for sex-specific markers (table 4). For screening and NHS health checks, broad code lists with standard lookback periods had the highest prevalence, whereas narrow code list with restrictive lookback had the lowest. For AAA screening and NHS health checks, changing the operational definition changed the prevalence <2% (online supplemental table 6). The prevalence of most markers was in line with national estimates, particularly for the vaccinations, PSA testing and bone density scans. For example, 73.3% of individuals in the current study had an influenza vaccination with national estimates reporting 72.4% influenza vaccination uptake among ≥65 years old in the 2019/2020 influenza vaccination season.^20^ The prevalence of screening and NHS health checks in the overall population was lower than national estimates, although this generally improved in comparison to currently eligible age-groups (figure 1). Hospital visit for an ACS condition were higher than literature estimates as it was not possible to differentiate planned and unplanned hospitalisations in the current data sets (9.5% in the current study vs 0.1% in literature).

The prevalence of markers typically varied by age category, with a number of patterns evident (figure 1). The recorded prevalence of markers with upper age eligibility (screening and NHS health checks) decreased with age (eg, 28.0% in 65–69 years old vs 1.5% in 85–89 years old for NHS health checks), whereas the prevalence of ACS conditions, blood pressure measurements and vaccinations rose with age (eg, 62.9% in 65–69 years old vs 80.5% in 85–89 years old for influenza vaccination). Although more common in younger age groups, screening marker prevalence still fell short of national estimates in currently eligible age-groups (eg, 54.3% in 65–69 years old vs 76.1% in national estimates for AAA screening in men). PSA tests, bone density scans, low-value procedures, low-value prescriptions and DNA primary care visits peaked at 75–89 years, with lower prevalence in younger and older individuals. GP visits were consistent across age categories. As expected, the proportion of individuals with ≥1 GP visit was very high. The post-hoc analysis revealed the number of GP visits increased by age category until the last age strata (90+ years), when it decreased slightly (online supplemental figure 3).

Using broad rather than narrow code lists, the estimated prevalences were similar for AAA screening across all age strata and for breast cancer screening in those aged 65–69 years. For all other breast cancer screening strata and for cervical cancer screening, broad code lists resulted in a higher prevalence than narrow. For standard versus restricted lookback periods, the prevalence was the same for individuals entering the cohort below the upper age of eligibility of that marker, whereas after this point there was a lower prevalence in the restricted versus standard age strata.

**Figure 2:**
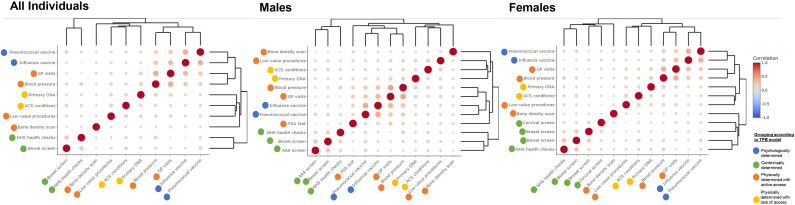
Correlation matrix plots. The correlations are calculated using phi coefficient for binary variables. The clustering is visualised through the adorned dendrograms which are ordered via complete linkage hierarchical clustering. The size and the shading of the bubble represent the strength of the correlation. Note: the correlation coefficients for these plots can be found in online supplemental tables 8–10. AAA, abdominal aortic aneurysm; ACS, ambulatory care sensitive; DNA, did not attend; GP, general practice; NHS, National Health Service; TPB, Theory of Planned Behaviour.

In the overall study population, unsurprisingly, GP visits were strongly correlated with blood pressure measurements (phi φ 0.42) and influenza vaccination (0.33). Blood pressure measurements were also strongly correlated with influenza vaccination (0.23). Markers with the strongest negative correlation were blood pressure measurements and NHS health checks (−0.14) (figure 2). Among men, GP visits and blood pressure measurements had the strongest positive correlation (0.45), followed by influenza and pneumococcal vaccinations (0.42). Other strong correlations included GP visits and pneumococcal vaccination (0.36) and blood pressure measurements and influenza vaccination (0.25) (figure 2). Among women, GP visits were also strongly correlated with blood pressure measurements (0.40) and blood pressure measurements with influenza vaccination (0.30). There were also strong correlations between pneumococcal vaccination with influenza vaccination (0.39), bowel cancer screening and NHS health checks (0.23) (figure 2).

Markers that were clustered together in the correlation matrices were: (1) blood pressure measurements, GP visits and influenza and pneumococcal vaccinations; (2) NHS health checks and bowel, cervical, breast cancer and AAA screening; and (3) ACS conditions, primary care DNA, bone density scans and low-value procedures. Markers from group 2 generally had a weak negative correlation with markers from group 3. When comparing these data-driven clusters with the theoretical grouping of markers there were some similarities. In both methods the ‘contextually determined’ (ie, NHS health checks and screenings) were grouped together as well as the ‘physically determined with a lack of healthcare access’ (ie, ACS conditions and primary care DNA). On the other hand, GP visits and blood pressure measurements were grouped with ‘psychologically determined’ markers in the data-driven approach, but with the ‘physically determined with active healthcare access’ in the theoretical grouping.

## Discussion

### A statement of the principal findings

Overall, this study found that it is feasible to identify markers of health-seeking behaviour and healthcare access in UK EHRs. The prevalence of these markers ranged significantly and were generally in line with national estimates. Screening and NHS health checks were under-recorded in the EHR data, although prevalence was closer to national estimates among younger age groups that were currently eligible for these programmes. The prevalence and pattern of markers differed by age, with AAA screening declining with older age and hospital visits for ACS condition increasing. Correlations between markers revealed clusters that aligned well with theoretical groupings informed by the updated TPB model based on psychological, contextual and physical underlying determinants.

### Strengths and weaknesses of the study

To our knowledge, this is the first study that has systematically identified proxies or markers of health-seeking behaviour or healthcare access using routinely collected data in the UK. Previous studies have adjusted for variables that may reflect confounding by health-seeking behaviour such as GP consultations, but without an explicit framework for selecting these. Our study demonstrates that a framework is beneficial since health-seeking behaviour and healthcare access are complex phenomena with multiple determinants, which may behave differently and vary by age and sex. Due to the number of different determinants that influence healthcare utilisation according to the updated TPB model, markers cannot solely be thought to solely represent either health-seeking behaviour or healthcare access. Linkage across primary and secondary care also strengthened this study as different types of healthcare utilisation with different underlying determinants could be captured. We included a large and representative cohort of over 2 million individuals aged 66 years and over in England. For some of the markers, older individuals might not have been historically eligible for services, which represents an important caveat during the interpretation of prevalence estimates. We accounted for this by calculating age-stratified prevalence, but for some of the prevalence comparisons the numerator and denominator populations need to be considered. For example, for NHS health checks the national estimate is of eligible individuals only (ie, those without pre-existing conditions^21^), whereas our denominator included all individuals aged ≥66 years in English.

The study also only measured markers of health-seeking behaviour and healthcare access at a single point in time: these characteristics are not static, and individual behaviour and service accessibility can change over time. In addition, for some of the identified markers the influence of health needs could not be entirely separated from health-seeking behaviour and healthcare access and therefore in some cases prevalence would be driven to some extent by health needs. These findings might also not be generalisable to younger individuals where perhaps there are other contextual determinants to consider (eg, occupation).^9^

### Strengths and weaknesses in relation to other studies/discussing important differences in results

Previous studies that have used EHR to identify markers of health-seeking behaviour and healthcare access in the USA^6^,^8^ are in a considerably different context from the UK in terms of health services provided (eg, screening programmes might differ), the healthcare system, claims-based recording systems and underlying determinants of health. This is likely to explain the different prevalence of markers identified in the current study. For example, the prevalence of pneumococcal vaccination was only around 11.4% in a study of ≥65 years old identified in the Medicare database with an influenza vaccination during the 2019/2020 season,^7^ whereas the prevalence was 62.4% in the current study. Screening coverage differs significantly between countries, for example, a global repository for breast, cervical and colorectal screening programmes reported that breast cancer screening coverage ranged from 1.7% in Bangladesh to 85.5% in the UK. For cervical cancer, coverage ranged from 2.1% in Côte d’Ivoire to 86.3% in Sweden and for colorectal cancer coverage ranged from 0.6% in Hungary to 64.5% in the Netherlands.^22^ Some countries might not even offer these preventative measures as free healthcare services or may offer them at different time points. In addition, within countries, some markers might change in how they adhere to the developed criteria over time or in different settings. For example, in the UK NICE guidance has recommended FIT tests for symptomatic testing since 2022.^17^ These differences support the importance of context-specific markers of health-seeking behaviour and healthcare access.

Our study adds to a growing body of literature highlighting the potential to capture proxies of healthcare access and health-seeking behaviour. In prior studies in the USA, these proxies were included as confounders during the estimation of vaccine effectiveness and they could play a similar role during observational studies in a UK context.^7^

### The meaning of the study: possible explanations and implications for researchers, clinicians and policymakers

The correlations between markers in the data are likely dictated by co-occurrence of these healthcare interactions (eg, blood pressure measurements might be taken when influenza vaccinations are administered) or influenced by individual patient characteristics (eg, age, underlying health conditions). Positive correlations (eg, the high positive correlation between AAA screening and bowel cancer screening) is likely because the prevalence of both these markers are highest among 65–69 years old with declining prevalence with age (figure 1). Negative correlations (eg, the high negative correlation between NHS health checks and influenza vaccinations) are likely due to underlying health conditions. For example, NHS health checks are offered to those without a list of pre-existing conditions,^21^ whereas the majority of the same conditions would qualify an individual for influenza vaccination prioritisation.^23^ This aligns with expectations according to the updated TPB model.^5^

Based on the findings presented here, we propose several recommendations and considerations for researchers that wish to identify health-seeking behaviour and healthcare access in EHRs—whether to study healthcare use directly, or to quantify or adjust for confounding.

First, a range of different markers are required to fully represent both active health-seeking behaviour and healthcare access, or lack of these. Since health-seeking behaviour/healthcare access is such a complex phenomenon, it may be useful to include markers with different underlying determinants from the updated TPB model (psychologically, contextually and physically determined). If multiple markers are available, they can be included as separate confounders in multivariate models, or researchers may wish to consider tools such as high-dimensional propensity scores to guide study-specific confounder identification, prioritisation and adjustment.^24^

Second, the optimal code lists will depend on the precise research question. Narrow code lists (eg, using government incentivised code lists) can identify markers of health-seeking behaviour and healthcare access with high specificity. Broader code lists will capture more events, but may be more influenced by underlying health needs. For markers with specific age-eligibility (eg, screening or NHS health checks) look-back periods that restrict to time periods when individuals were age eligible improved specificity. However, more relaxed lookback periods might be preferred if there are expected to be artefacts in data recording such as transfer of historical information to EHRs.

Third, prior to adjusting for health-seeking behaviour and healthcare access, interactions by age, sex and underlying health conditions should be considered. Markers that were recently introduced into clinical practice (eg, AAA screening was introduced in the UK in 2013^25^) will likely decrease in prevalence with increasing age and can be supplemented with markers that increase with increasing age (eg, ACS conditions). Otherwise, markers with relatively consistent prevalence across age strata are available (eg, GP visits or blood pressure measurements). If markers that are restricted to specific sex (eg, breast cancer screening) are used then these can be supplemented with markers of the opposite sex (eg, AAA screening). For markers where there is some partial influence of underlying health conditions (eg, pneumococcal vaccinations recommended to all but may be more highly prioritised among those with high-risk conditions) can be supplemented with markers that are administered to those that are healthier (eg, NHS health checks).

### Unanswered questions and future research

Future researchers who are concerned with potential confounding from health-seeking behaviour and healthcare access in their study can use these markers to quantify and adjust for confounding. Where possible, a range of markers with different underlying determinants from the updated TPB model should be used and possible interactions by age, sex and underlying condition should be considered. Future research may identify key confounders within each theoretical group or cluster that are sufficient for confounding adjustment, although these are likely to be study-specific.

Common data models across data sets could increase the efficiency and comparability of research investigating or adjusting for health-seeking behaviour and healthcare access, but future research is needed to identify suitable markers in alternative data sets and establish comparability. Additional markers may be identified in alternative data sets using the developed criteria.

### Conclusion

Overall, markers of health-seeking behaviour and healthcare access can be identified in UK EHR, with prevalence estimates in line with national estimates. National screening programme estimates still fell short of national estimates even when restricting to currently eligible age groups. The generally low correlations between different proxy markers of health-seeking behaviour and healthcare access, and different age-profiles of markers, suggest a range of variables are needed to capture different determinants of healthcare use.

## supplementary material

10.1136/bmjopen-2023-081781 online supplemental file 1

## Data Availability

Data may be obtained from a third party and are not publicly available.

## References

[1] Kasl SV, Cobb S (1966). Health behavior, illness behavior and sick role behavior. Arch Environ Health: Int J.

[2] University of Missouri (2023). Health care access. https://medicine.missouri.edu/centers-institutes-labs/health-ethics/faq/health-care-access#:~:text=Health%20care%20access%20is%20the,and%20other%20health%2Dimpacting%20conditions.

[3] Jackson LA, Jackson ML, Nelson JC (2006). Evidence of bias in estimates of influenza vaccine effectiveness in seniors. *Int J Epidemiol*.

[4] English Longitudinal Study of Ageing (2023). The data we collect. https://www.elsa-project.ac.uk/the-data-we-collect.

[5] Schmid P, Rauber D, Betsch C (2017). Barriers of influenza vaccination intention and behavior - a systematic review of influenza vaccine hesitancy, 2005 - 2016. PLoS ONE.

[6] Izurieta HS, Chillarige Y, Kelman J (2020). Relative effectiveness of influenza vaccines among the United States elderly, 2018-2019. J Infect Dis.

[7] Izurieta HS, Lu M, Kelman J (2021). Comparative effectiveness of influenza vaccines among US medicare beneficiaries ages 65 years and older during the 2019-2020 season. Clin Infect Dis.

[8] Zhang HT, McGrath LJ, Wyss R (2017). Controlling confounding by frailty when estimating influenza vaccine effectiveness using predictors of dependency in activities of daily living. *Pharmacoepidemiol Drug Saf*.

[9] Cowling TE, Ramzan F, Ladbrooke T (2016). Referral outcomes of attendances at general practitioner led urgent care centres in London, England: retrospective analysis of hospital administrative data. *Emerg Med J*.

[10] Salive ME (2013). Multimorbidity in older adults. Epidemiol Rev.

[11] NHS Digital Unplanned hospitalisation for chronic ambulatory care sensitive conditions.

[12] The National Institute for Health and Care Excellence (2022). NICE “do not do” recommendations. https://www.nice.org.uk/media/default/sharedlearning/716_716donotdobookletfinal.pdf.

[13] NHS England (2023). Items which should not be routinely prescribed in primary care.

[14] Williams R, Kontopantelis E, Buchan I (2017). Clinical code set engineering for reusing EHR data for research: A review. J Biomed Inform.

[15] NHS Digital (2023). Quality and Outcomes Framework (QOF).

[16] UK Government Population screening programmes: bowel cancer.

[17] National Institute for Health and Care Excellence (2024). Quantitative faecal immunochemical tests to guide colorectal cancer pathway referral in primary care.

[18] UK Government (2022). Population screening programmes: breast cancer.

[19] Mukaka MM (2012). Statistics corner: A guide to appropriate use of correlation coefficient in medical research. *Malawi Med J*.

[20] UK Government (2023). Fingertips, public health data, population vaccination coverage: Flu (aged 65 and older).

[21] National Health Service (2024). NHS Health Checks. https://www.nhs.uk/conditions/nhs-health-check/.

[22] Zhang L, Mosquera I, Lucas E (2023). CanScreen5, a global repository for breast, cervical and colorectal cancer screening programs. *Nat Med*.

[23] UK Government (2022). Greenbook chapter 19. https://assets.publishing.service.gov.uk/government/uploads/system/uploads/attachment_data/file/931139/Green_book_chapter_19_influenza_V7_OCT_2020.pdf.

[24] Tazare J, Wyss R, Franklin JM (2022). Transparency of high-dimensional propensity score analyses: Guidance for diagnostics and reporting. *Pharmacoepidemiol Drug Saf*.

[25] The Health Foundation (2023). The Abdominal Aortic Aneurysm (AAA) screening programme.

[26] Herbert A, Wijlaars L, Zylbersztejn A (2017). Data resource profile: hospital episode statistics admitted patient care (HES APC). Int J Epidemiol.

[27] Clinical Practice Research Datalink (2023). Release notes: cprd aurum may 2022. https://cprd.com/sites/default/files/2022-05/2022-05%20CPRD%20Aurum%20Release%20Notes.pdf.

[28] Office for National Statistics (2024). Estimates of the population for the uk, england, wales, scotland, and northern ireland. https://www.ons.gov.uk/peoplepopulationandcommunity/populationandmigration/populationestimates/datasets/populationestimatesforukenglandandwalesscotlandandnorthernireland.

[29] UK Government (2022). Population screening programmes: abdominal aortic aneurysm.

[30] UK Government (2023). Cervical screening: programme overview.

[31] UK Government (2023). NHS health checks: applying all our health.

[32] UK Government (2022). Greenbook chapter 25. https://assets.publishing.service.gov.uk/government/uploads/system/uploads/attachment_data/file/674074/GB_Chapter_25_Pneumococcal_V7_0.pdf.

[33] NHS England (2023). PSA testing.

[34] NHS England (2023). Bone density (DEXA scan).

[35] NHS Digital (2023). Appointments in general Practice report.

[36] Watt T, Sullivan R, Aggarwal A (2022). Primary care and cancer: an analysis of the impact and inequalities of the COVID-19 pandemic on patient pathways. *BMJ Open*.

[37] NHS England Emergency admissions for ambulatory care sensitive conditions – characteristics and trends at national level 2023.

[38] NHS England (2023). Blood pressure test.

