# Supplementary material for: Identifying markers of health-seeking behaviour and healthcare access in UK electronic health records
Source: BMJ Open. 2024 Sep 26;14(9):e081781. doi: 10.1136/bmjopen-2023-081781 (PMC11429345; doi:10.1136/bmjopen-2023-081781)
Supplement: online supplemental file 1 [file bmjopen-14-9-s001.pdf]

Supplementary information
Tables

Supplementary Table 1. Current available literature and markers derived from each

Author and year	Study type	Markers identified	Included/excluded	Reason for exclusion
Izurieta et al, 2020[1]	Observational cohort study of relative influenza vaccine effectiveness using US claims data	Pneumococcal vaccination	Included	
		Annual wellness visit	Included as NHS health checks	
		Bone mass measurements	Included as bone density scan	
		Cardiovascular disease screen tests	Excluded	Failed criteria 3
		Colorectal cancer screen	Included as bowel cancer screen	
		Diabetes screen	Included as NHS health checks	
		Initial Preventive Physical Examination	Included as NHS health checks	
		Prostate Cancer Screen	Included as PSA test	
		Screen Mammography	Included as breast cancer screen	
		Screen Pap test	Included as cervical cancer screen	
		Screen pelvic examination	Excluded	Failed criteria 3
		Depression screen	Excluded	Failed criteria 3
		Other preventative services	Excluded	Unclear what this entails
Izurieta et al, 2021[2]	Observational cohort study of influenza vaccine effectiveness using US claims data	Annual Wellness Visits	Included as NHS health checks	
		Counseling & Health Risk Assessment	Excluded	Failed criteria 3
		Pneumococcal Vaccination	Included	
		Tetanus-containing vaccination	Excluded	Failed criteria 3
		Shingrix vaccination	Excluded	Failed criteria 1
Zhang et al, 2017[3]	Observational cohort study of influenza vaccine effectiveness using US claims data	Outpatient visits	Included as GP visits	
		Hospital visits	Excluded	Failed criteria 3
		Colonoscopies	Included as bowel cancer screen	
		Fecal occult blood tests	Included as bowel cancer screen	

Abbreviations: DNA: did not attend; GP: general practice; NHS: national health service; PSA: prostate-specific antigen.

Supplementary Table 2. How each of the markers meet each of the criteria

Marker	**Criteria 1**<br>Should have been currently or recently available in national clinical practice to all individuals (overall or by sex) at cohort entry.	**Criteria 2**<br>Should be routinely recorded in the available data sources.	**Criteria 3**<br>Should not be primarily dependent on underlying health needs.
AAA screen	~<br><br>AAA screening was introduced in 2013 in the UK[4] so for men entering the cohort at age 71 years and above they might not have ever been offered a screen (since this is offered to all men when they turn 65 years).	✓	✓<br><br>Universally available except for men that have been treated for an AAA previously. The incidence of AAA in those under 65 years is very low[5].
Breast cancer screen	✓	✓	✓

Marker	**Criteria 1** Should have been currently or recently available in national clinical practice to all individuals (overall or by sex) at cohort entry.	**Criteria 2** Should be routinely recorded in the available data sources.	**Criteria 3** Should not be primarily dependent on underlying health needs.
Cervical cancer screen	✓	✓	✓
Bowel cancer screen	✓	✓	✓
NHS health checks	✓	✓	~ Universally available to those without pre-existing high-risk conditions[6].
Influenza vaccination	✓	✓	✓
Pneumococcal vaccination	✓	✓	✓
PSA testing	✓	✓	~ Men can request a PSA test with no prior diagnosis of prostate cancer who are asymptomatic, men presenting with symptoms, and men who have had a previous high PSA level and are being monitored[7].
Bone density scans	✓	✓	~ Can be requested for individuals over 50 years with a risk of developing osteoporosis or for those with other risk factors such as smoking or broken bone[8].
GP practice visits	✓	✓	~ Occurs for those with symptoms.
Low-value procedures	✓	✓	~ Requested for those with symptoms.
Low-value prescriptions	✓	~ Although these can be prescribed in primary care, individuals can also buy some low values prescriptions over the counter and therefore we might see under recording of this. However, those that access primary care for something that can be bought over the counter likely have very active access to healthcare.	✓
Hospital visit for an ACS condition	✓	✓	~ Occurs for those with symptoms.
DNA primary care visit	✓	✓	~ Occurs for those with symptoms.
Blood pressure measurements	✓	~	~ Universally available, but disproportionately requested for those with underlying health conditions.

Abbreviations: DNA: did not attend; GP: general practice; NHS: national health service; PSA: prostate-specific antigen.

Supplementary Table 3. Influenza Vaccination Algorithm

Combination of codes on same day	Total number of vaccination events	Decision	Rationale
Given and neutral	725432	Record as valid vaccination.	
Given and absent	1149 (of these only 924 are the first vaccination dose)	Do not record as valid vaccination.	The prevalence of this marker may be underestimated very slightly, however, in this instance better to be more specific than sensitive when it comes to confounders[9].
Given and adverse	14	Record as valid vaccination.	Likely that this patient received the vaccination, but then had an adverse event on the same day.
Given and product	395881	Record as valid vaccination.	
Given and given with lag	4700	Record as valid vaccination.	
Neutral and absent	1178	Do not record as valid vaccination.	
Neutral and adverse	9	Record as valid vaccination.	
Neutral and product	295746	Record as valid vaccination.	
Neutral and given lag	7748	Record as valid vaccination.	
Absent and adverse	27	Do not record as valid vaccination.	Likely that these patients are reporting a previous adverse event as the reason for not wanting to get vaccinated.
Absent and product	218 (of these only 194 are first vaccination dose)	Do not record as valid vaccination.	The prevalence of this marker will be underestimated very slightly, however, in this instance better to be more specific than sensitive when it comes to confounders[9].
Absent and given with lag	570	Record as valid vaccination.	This most likely reflects that the reason a vaccine wasn't given on the event date is that the patient had already had it elsewhere. We can be reasonably confident that the patient was vaccinated, but we don't know the exact date.
Adverse and product	1	Record as valid vaccination.	Likely that this patient received the vaccination, but then had an adverse event on the same day.
Adverse and given with lag	0	Ignore – no events.	
Given with lag and product	784	Record as valid vaccination.	

Note: since influenza vaccinations can be identified using both medcodes and prodcodes and since medcodes do not always insinuate presence of a vaccination, an algorithm was developed for combinations of codes that occurred on the same day. Medcodes were separated into those that were clearly given ("given" or "administered"), given with delay ("given" or "administered" but evidence this occurred in another setting previously), neutral (vaccination mentioned but no "given" or "administered") and absent (vaccination "refused" or "not consented). Then we looked at vaccination events (using both medcodes and prodcodes) that were recorded on the same date and categorised these according to the above framework.

Supplementary Table 4. National estimates

Marker	Estimate (%, unless otherwise specified)	Year	Numerator	Denominator
AAA screen[10]	76.1	2019/20	Number of men eligible for the initial screen who have had a conclusive scan result within the screening year plus an additional 3 months (in the event of non attendance and cancellations at the end of the year this allows men to be reinvited and screened).	Number of eligible men in their 65th year to whom the screening programme propose that a screening encounter during the reporting period should be offered.
Breast cancer screen[11]	71.1	2019/20	The number of persons registered to the practice who were screened adequately in the previous 36 months	The number of eligible persons on last day of the review period
Cervical cancer screen[12]	76.2	2019	The number of women in the resident population eligible for cervical screening aged 50 to 64 years at end of period reported who were screened adequately within the previous 5.5 years.	The number of women in the resident population eligible for cervical screening aged 50 to 64 years
Bowel cancer screen[13]	60.5	2019	Adequately screened (numerator) is the number of eligible men and women who have had an adequate gFOBT screening result recorded in the past 30 months.	Eligible population (denominator) is the number of men and women aged 60 to 74 years resident in the area (determined by postcode of residence) who are eligible for bowel cancer screening at a given point in time, excluding those whose recall has been ceased for clinical reasons (e.g. no functioning colon) or if they opt out of the programme.

NHS health checks[14]	2.0	2019/20 Q1	Number of people aged 40-74 eligible for an NHS Health Check who were recorded as receiving an NHS Health Check in the current quarter	Number of people aged 40-74 eligible for an NHS Health Check in the financial year.
Influenza vaccination[15]	72.4	2019/20	The number of adults aged 65 and over, who received the flu vaccination between 1st September to the end of February as recorded in the GP record.	Number of adults aged 65 years and older
Pneumococcal vaccination[16]	69	2019/20	These data describe pneumococcal polysaccharide vaccine (PPV) uptake for the survey year, for those aged 65 years and over.	Those aged 65 years and over
PSA test[17]	52.95	2002-2011	Number of men with at least one PSA test identified over a 10 year period	Number of men
Bone density scan[18]	0.3 to 16.2 per 1000 weighted population	2013/14	Number of bone scans.	Total population.
GP practice visits[19]	39127 per 100 000 patient-months	2019	Number of primary care consultations in CPRD Aurum	Number of patient months
GP practice visits[19]	26 919 consultations per 100 000 patient-months	2020	Number of primary care consultations in CPRD Aurum	Number of patient months
DNA primary care visit[19]	Number: 23578484	Dec-19	Number of primary care appointments that were DNA	
Low value procedures[20]	0.02-0.2	2017/18	Number of category 1 interventions.	Total English population, age and sex-standardised.
Low-value prescriptions (glucosamine)	21,961 items	2019/20	Total quantity prescribed in primary care	
Hospital visit for an ACS condition[21]	3-15%	2012/13	Total number of individuals with a hospital admission for an unplanned hospital admission for an ACS condition	Total population in age and gender specific bands
Blood pressure test[22]	84.6	2012-13	Number of individuals aged ≥50 years with a blood pressure check in the last year	Number of individuals aged ≥50 years

Abbreviations: DNA: did not attend; GP: general practice; NHS: national health service; PSA: prostate-specific antigen.

Supplementary Table 5. Study population.

**Group**	**Category**	**All N= 1,991,284**
Age continuous, mean (SD)		75.9 (7.4)
Age category, N (%)	65-69	448,063 (22.5%)
	70-74	557,599 (28.0%)
	75-79	401,290 (20.2%)
	80-84	295,492 (14.8%)
	85-89	181,835 (9.1%)
	90-95	80,943 (4.1%)
	95+	26,062 (1.3%)
Sex, N (%)	Female	1,075,723 (54.0%)
Ethnicity, N (%)	Asian	67,961 (3.4%)
	Black	36,912 (1.9%)
	Mixed	10,072 (0.5%)
	Other	17,711 (0.9%)
	White	1,759,754 (88.4%)
	Missing	98,874 (5.0%)
Region, N (%)	East Midlands	42,106 (2.1%)
	East of England	95,413 (4.8%)
	London	263,825 (13.2%)
	North East	67,278 (3.4%)

	North West	381,592 (19.2%)
	South East	438,407 (22.0%)
	South West	270,484 (13.6%)
	West Midlands	357,363 (17.9%)
	Yorkshire and The Humber	74,805 (3.8%)
	Unknown	11 (0.0%)

Abbreviations: N: number; SD: standard deviation.

Supplementary Table 6. Prevalence of markers using different definitions

**Variable**	**All**	**Male**	**Female**
N	1,991,284	915,561	1,075,723
AAA screen	231,088 (11.6%)	227,844 (24.9%)	3,244 (0.3%)
AAA screen broad*	238,186 (12.0%)	233,574 (25.5%)	4,612 (0.4%)
Breast cancer screen restrictive†	253,610 (12.7%)	227 (0.0%)	253,383 (23.6%)
Breast cancer screen	346,116 (17.4%)	517 (0.1%)	345,599 (32.1%)
Breast cancer screen broad* restrictive†	484,402 (24.3%)	573 (0.1%)	483,829 (45.0%)
Breast cancer screen broad*	686,521 (34.5%)	1,517 (0.2%)	685,004 (63.7%)
Cervical cancer screen restrictive†	256,565 (12.9%)	40 (0.0%)	256,525 (23.8%)
Cervical cancer screen	397,303 (20.0%)	153 (0.0%)	397,150 (36.9%)
Cervical cancer screen broad* restrictive†	373,369 (18.8%)	93 (0.0%)	373,276 (34.7%)
Cervical cancer screen broad*	588,288 (29.5%)	361 (0.0%)	587,927 (54.7%)
NHS health checks restrictive†	338,441 (17.0%)	143,325 (15.7%)	195,116 (18.1%)
NHS health checks	372,244 (18.7%)	157,484 (17.2%)	214,760 (20.0%)
Bowel screen restrictive†	1,151,943 (57.8%)	553,109 (60.4%)	598,834 (55.7%)
Bowel screen	1,439,412 (72.3%)	687,712 (75.1%)	751,700 (69.9%)

Abbreviations: AAA: abdominal aortic aneurysm; DNA: did not attend; GP: general practice; PSA: prostate-specific antigen.
*Broad code list: code lists were less specific e.g., screening markers could mention the relevant test, but without requiring “screen” in the code.
†Restrictive lookback: for markers with an upper age of eligibility (e.g., cancer screening and NHS health checks) the lookback period stopped at the age of upper eligibility.

Supplementary Table 7. Prevalence of markers by age.

Marker	Age category	Count	Prevalence
AAA screen broad*	65-69	121,345	55.0
AAA screen broad*	70-74	87,776	32.7
AAA screen broad*	75-79	13,957	7.4
AAA screen broad*	80-84	6,930	5.3
AAA screen broad*	85-89	2,748	3.7
AAA screen broad*	90-95	709	2.5
AAA screen broad*	95+	109	1.6
AAA screen	65-69	119,707	54.3
AAA screen	70-74	85,529	31.8
AAA screen	75-79	13,075	7.0
AAA screen	80-84	6,360	4.9
AAA screen	85-89	2,445	3.3
AAA screen	90-95	634	2.3
AAA screen	95+	94	1.4
Breast cancer screen broad*	65-69	165,587	72.8
Breast cancer screen broad*	70-74	214,147	74.1
Breast cancer screen broad*	75-79	160,055	74.9
Breast cancer screen broad*	80-84	104,683	63.5
Breast cancer screen broad*	85-89	30,543	28.2
Breast cancer screen broad*	90-95	8,351	15.8
Breast cancer screen broad*	95+	1,638	8.5
Breast cancer screen broad* restrictive†	65-69	165,575	72.8
Breast cancer screen broad* restrictive†	70-74	163,148	56.4
Breast cancer screen broad* restrictive†	75-79	81,247	38
Breast cancer screen broad* restrictive†	80-84	62,039	37.6
Breast cancer screen broad* restrictive†	85-89	10,162	9.4
Breast cancer screen broad* restrictive†	90-95	1,500	2.8
Breast cancer screen broad* restrictive†	95+	158	0.8
Breast cancer screen	65-69	86,335	37.9
Breast cancer screen	70-74	112,561	38.9
Breast cancer screen	75-79	81,241	38
Breast cancer screen	80-84	48,422	29.4
Breast cancer screen	85-89	13,182	12.2
Breast cancer screen	90-95	3,300	6.2
Breast cancer screen	95+	558	2.9
Breast cancer screen restrictive†	65-69	86,327	37.9
Breast cancer screen restrictive†	70-74	87,674	30.3
Breast cancer screen restrictive†	75-79	44,831	21
Breast cancer screen restrictive†	80-84	29,008	17.6
Breast cancer screen restrictive†	85-89	4,817	4.4
Breast cancer screen restrictive†	90-95	679	1.3
Breast cancer screen restrictive†	95+	47	0.2
Cervical cancer screen broad*	65-69	127,792	56.2

Cervical cancer screen broad*	70-74	160,051	55.4
Cervical cancer screen broad*	75-79	122,449	57.3
Cervical cancer screen broad*	80-84	91,696	55.6
Cervical cancer screen broad*	85-89	57,058	52.7
Cervical cancer screen broad*	90-95	24,576	46.5
Cervical cancer screen broad*	95+	4,305	22.3
Cervical cancer screen broad* restrictive†	65-69	95,951	42.2
Cervical cancer screen broad* restrictive†	70-74	114,760	39.7
Cervical cancer screen broad* restrictive†	75-79	78,703	36.8
Cervical cancer screen broad* restrictive†	80-84	49,255	29.9
Cervical cancer screen broad* restrictive†	85-89	25,412	23.5
Cervical cancer screen broad* restrictive†	90-95	8,537	16.1
Cervical cancer screen broad* restrictive†	95+	658	3.4
Cervical cancer screen	65-69	76,059	33.4
Cervical cancer screen	70-74	112,676	39
Cervical cancer screen	75-79	85,484	40
Cervical cancer screen	80-84	63,964	38.8
Cervical cancer screen	85-89	39,581	36.6
Cervical cancer screen	90-95	16,649	31.5
Cervical cancer screen	95+	2,737	14.2
Cervical cancer screen restrictive†	65-69	59,641	26.2
Cervical cancer screen restrictive†	70-74	81,978	28.4
Cervical cancer screen restrictive†	75-79	55,434	25.9
Cervical cancer screen restrictive†	80-84	35,276	21.4
Cervical cancer screen restrictive†	85-89	17,966	16.6
Cervical cancer screen restrictive†	90-95	5,846	11.1
Cervical cancer screen restrictive†	95+	384	2.0
Bowel cancer screen	65-69	423,248	94.5
Bowel cancer screen	70-74	521,588	93.5
Bowel cancer screen	75-79	354,378	88.3
Bowel cancer screen	80-84	114,222	38.7
Bowel cancer screen	85-89	17,514	9.6
Bowel cancer screen	90-95	6,743	8.3
Bowel cancer screen	95+	1,719	6.6
Bowel cancer screen restrictive†	65-69	423,248	94.5
Bowel cancer screen restrictive†	70-74	513,796	92.1
Bowel cancer screen restrictive†	75-79	170,462	42.5
Bowel cancer screen restrictive†	80-84	42,108	14.3
Bowel cancer screen restrictive†	85-89	1,903	1.0
Bowel cancer screen restrictive†	90-95	391	0.5
Bowel cancer screen restrictive†	95+	35	0.1
NHS health checks	65-69	125,225	27.9
NHS health checks	70-74	135,076	24.2
NHS health checks	75-79	85,472	21.3
NHS health checks	80-84	22,626	7.7
NHS health checks	85-89	2,781	1.5

NHS health checks	90-95	844	1.0
NHS health checks	95+	220	0.8
NHS health checks restrictive†	65-69	125,225	27.9
NHS health checks restrictive†	70-74	134,622	24.1
NHS health checks restrictive†	75-79	69,649	17.4
NHS health checks restrictive†	80-84	8,933	3.0
NHS health checks restrictive†	85-89	6	<0.1
NHS health checks restrictive†	90-95	6	<0.1
NHS health checks restrictive†	95+	0	0
Influenza vaccination	65-69	281,993	62.9
Influenza vaccination	70-74	400,143	71.8
Influenza vaccination	75-79	310,417	77.4
Influenza vaccination	80-84	237,347	80.3
Influenza vaccination	85-89	146,427	80.5
Influenza vaccination	90-95	64,238	79.4
Influenza vaccination	95+	19,826	76.1
Pneumococcal vaccination	65-69	171,814	38.3
Pneumococcal vaccination	70-74	329,895	59.2
Pneumococcal vaccination	75-79	287,843	71.7
Pneumococcal vaccination	80-84	229,129	77.5
Pneumococcal vaccination	85-89	142,058	78.1
Pneumococcal vaccination	90-95	62,385	77.1
Pneumococcal vaccination	95+	19,235	73.8
PSA testing	65-69	71,613	32.5
PSA testing	70-74	101,779	37.9
PSA testing	75-79	79,356	42.3
PSA testing	80-84	56,678	43.4
PSA testing	85-89	30,517	41.5
PSA testing	90-95	10,108	36
PSA testing	95+	1,833	27.2
Bone density scans	65-69	19,797	4.4
Bone density scans	70-74	29,459	5.3
Bone density scans	75-79	23,462	5.8
Bone density scans	80-84	16,393	5.5
Bone density scans	85-89	8,844	4.9
Bone density scans	90-95	2,544	3.1
Bone density scans	95+	393	1.5
GP visits	65-69	396,941	88.6
GP visits	70-74	511,875	91.8
GP visits	75-79	377,556	94.1
GP visits	80-84	281,987	95.4
GP visits	85-89	174,447	95.9
GP visits	90-95	77,495	95.7
GP visits	95+	24,522	94.1
DNA primary care	65-69	118,146	26.4
DNA primary care	70-74	151,958	27.3

DNA primary care	75-79	124,316	31
DNA primary care	80-84	102,601	34.7
DNA primary care	85-89	67,750	37.3
DNA primary care	90-95	29,068	35.9
DNA primary care	95+	8,057	30.9
Low value procedures	65-69	59,141	13.2
Low value procedures	70-74	87,714	15.7
Low value procedures	75-79	77,838	19.4
Low value procedures	80-84	66,686	22.6
Low value procedures	85-89	43,871	24.1
Low value procedures	90-95	18,545	22.9
Low value procedures	95+	5,086	19.5
Low value prescriptions	65-69	29	<0.1
Low value prescriptions	70-74	58	<0.1
Low value prescriptions	75-79	57	<0.1
Low value prescriptions	80-84	40	<0.1
Low value prescriptions	85-89	22	<0.1
Low value prescriptions	90-95	<5	<0.1
Low value prescriptions	95+	<5	<0.1
Hospital visit ACS condition	65-69	23,948	5.3
Hospital visit ACS condition	70-74	36,985	6.6
Hospital visit ACS condition	75-79	36,772	9.2
Hospital visit ACS condition	80-84	37,255	12.6
Hospital visit ACS condition	85-89	30,822	17
Hospital visit ACS condition	90-95	17,519	21.6
Hospital visit ACS condition	95+	6,835	26.2
Blood pressure measurement	65-69	288,444	64.4
Blood pressure measurement	70-74	394,102	70.7
Blood pressure measurement	75-79	307,207	76.6
Blood pressure measurement	80-84	240,202	81.3
Blood pressure measurement	85-89	151,973	83.6
Blood pressure measurement	90-95	67,185	83
Blood pressure measurement	95+	20,893	80.2

Abbreviations: AAA: abdominal aortic aneurysm; ACS: ambulatory care sensitive; DNA: did not attend; GP: general practice; NHS: National Health Service; PSA: prostate-specific antigen.

*Broad code list: code lists were less specific e.g., screening markers could mention the relevant test, but without requiring “screen” in the code.

†Restrictive lookback: for markers with an upper age of eligibility (e.g., cancer screening and NHS health checks) the lookback period stopped at the age of upper eligibility.

Supplementary Table 8. Phi coefficients

	NHS health checks	Bone density scan	Bowel screen	Primary DNA	Blood pressure	Pneumococcal vaccine	Influenza vaccine	ACS conditions	Low-value procedures	GP visits
NHS health checks	1									
Bone density scan	0.02	1								
Bowel screen	0.21	0.02	1							
Primary DNA	-0.06	0.03	-0.05	1						
Blood pressure	-0.14	0.03	-0.08	0.16	1					
Pneumococcal vaccine	-0.06	0.03	-0.12	0.07	0.18	1				
Influenza vaccine	-0.01	0.04	-0.03	0.06	0.23	0.41	1			
ACS conditions	-0.08	0.02	-0.12	0.11	0.13	0.07	0.05	1		
Low-value procedures	-0.05	0.04	-0.06	0.12	0.11	0.07	0.06	0.15	1	
GP visits	0.01	0.05	-0.02	0.14	0.42	0.21	0.33	0.07	0.11	1

Abbreviations: ACS: ambulatory care sensitive; DNA: did not attend; GP: general practice; NHS: National Health Service.
Note: the correlations are calculated using phi coefficient for binary variables.

Supplementary Table 9. Phi coefficients males

	AAA screen	NHS health checks	PSA test	Bone density scan	Bowel screen	Primary DNA	Blood pressure	Pneumococcal vaccine	Influenza vaccine	ACS conditions	Low-value procedures	GP visits
AAA screen	1											
NHS health checks	0.11	1										
PSA test	0.00	0.03	1									
Bone density scan	-0.01	0.00	0.06	1								
Bowel screen	0.24	0.17	0.01	-0.01	1							
Primary DNA	-0.05	-0.05	0.06	0.03	-0.05	1						
Blood pressure	-0.04	-0.14	0.12	0.03	-0.06	0.17	1					
Pneumococcal vaccine	-0.12	-0.07	0.1	0.03	-0.12	0.07	0.22	1				

Influenza vaccine	-0.02	-0.02	0.13	0.03	-0.05	0.07	0.26	0.42	1			
ACS conditions	-0.07	-0.07	0.02	0.03	-0.11	0.11	0.1	0.07	0.06	1		
Low-value procedures	-0.04	-0.04	0.07	0.04	-0.06	0.12	0.12	0.07	0.07	0.16	1	
GP visits	0.01	0.00	0.17	0.04	-0.02	0.15	0.45	0.23	0.36	0.07	0.11	1

Abbreviations: AAA: abdominal aortic aneurysm; ACS: ambulatory care sensitive; DNA: did not attend; GP: general practice; NHS: National Health Service; PSA: prostate-specific antigen.
Note: the correlations are calculated using phi coefficient for binary variables.

Supplementary Table 10. Phi coefficients females

	Breast screen	Cervical screen	NHS health checks	Bone density scan	Bowel screen	Primary DNA	Blood pressure	Pneumococcal vaccine	Influenza vaccine	ACS conditions	Low-value procedures	GP visits
Breast screen	1											
Cervical screen	0.11	1										
NHS health checks	0.09	0.05	1									
Bone density scan	0.03	0.01	0.03	1								
Bowel screen	0.21	0.06	0.23	0.04	1							
Primary DNA	-0.02	-0.02	-0.06	0.03	-0.05	1						
Blood pressure	0.00	0.02	-0.14	0.03	-0.09	0.16	1					
Pneumococcal vaccine	0.01	0.06	-0.06	0.03	-0.11	0.07	0.17	1				
Influenza vaccine	0.05	0.06	0.00	0.04	-0.02	0.05	0.21	0.39	1			
ACS conditions	-0.05	-0.03	-0.08	0.01	-0.12	0.11	0.12	0.06	0.04	1		
Low-value procedures	-0.01	0.00	-0.05	0.05	-0.05	0.11	0.11	0.07	0.05	0.15	1	
GP visits	0.03	0.05	0.01	0.05	-0.02	0.14	0.41	0.19	0.31	0.07	0.10	1

Abbreviations: ACS: ambulatory care sensitive; DNA: did not attend; GP: general practice; NHS: National Health Service.
Note: The correlations are calculated using phi coefficient for binary variables.

Figures

Supplementary Figure 1. Demonstration of lookback periods for markers with upper age of eligibility. For markers with an upper age of eligibility (breast, cervical, bowel cancer screening and NHS health checks), if an individual was aged under the upper age limit at index, then we used a consistent lookback period (e.g., three years). For individuals aged over this limit at index, the lookback period would start at a consistent age (e.g., 67 years) and would extend until their index date. This decision was taken as there was a concern that for older individuals electronic data transfer would have meant that the date of transfer was recorded rather than the date of event occurrence.

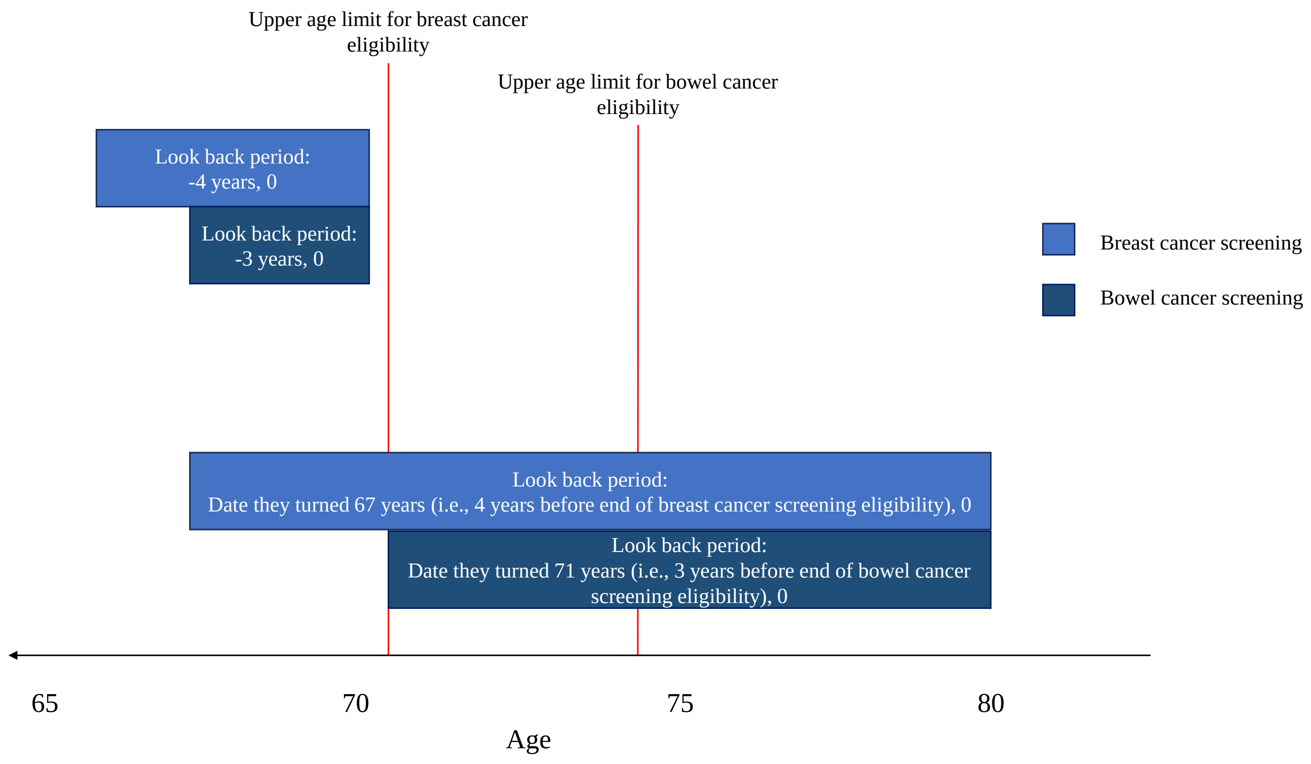

Supplementary Figure 2. Histogram of total number of GP visits per patient in one year before index. As majority of individuals in the study had at least one GP visit, we conducted a *post-hoc analysis* that identified the total number of GP visits per patient.

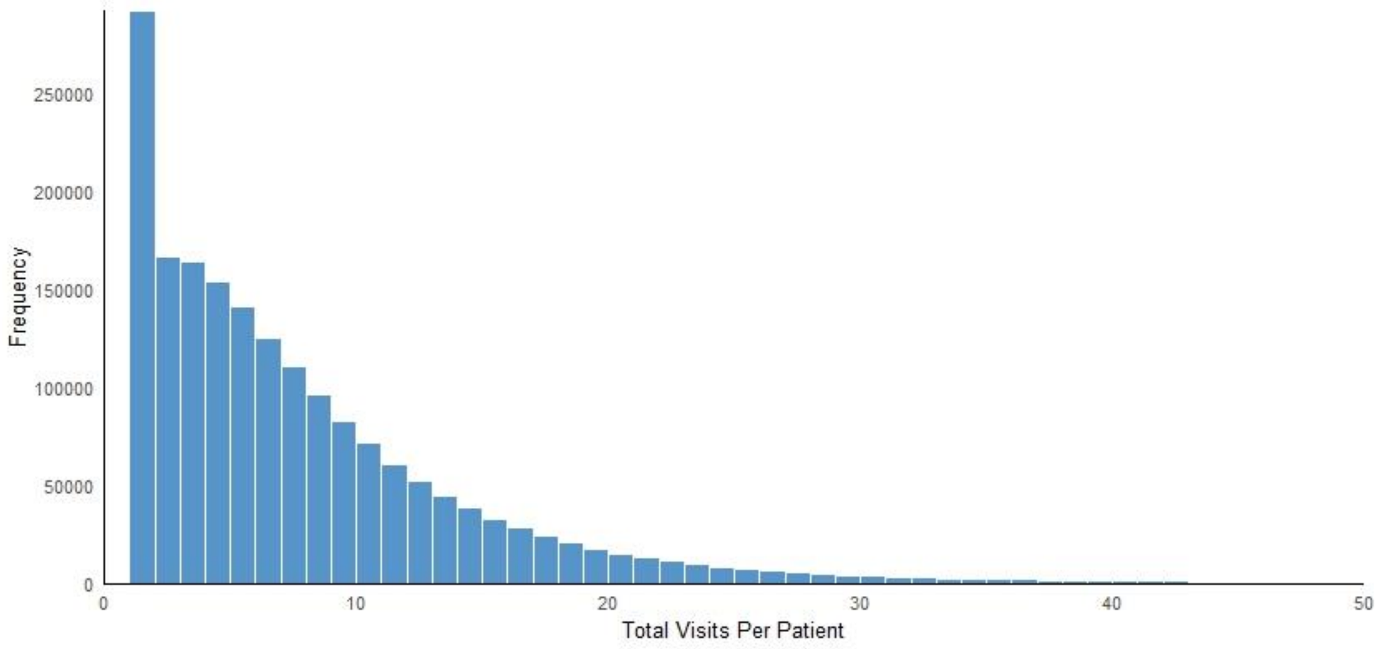

Supplementary Figure 3: Prevalence of over the median number of GP visits, stratified by age category. As majority of individuals in the study had at least one GP visit, we conducted a *post-hoc analysis* that identified the prevalence of over the median number of visits per year (7), stratified by age category.

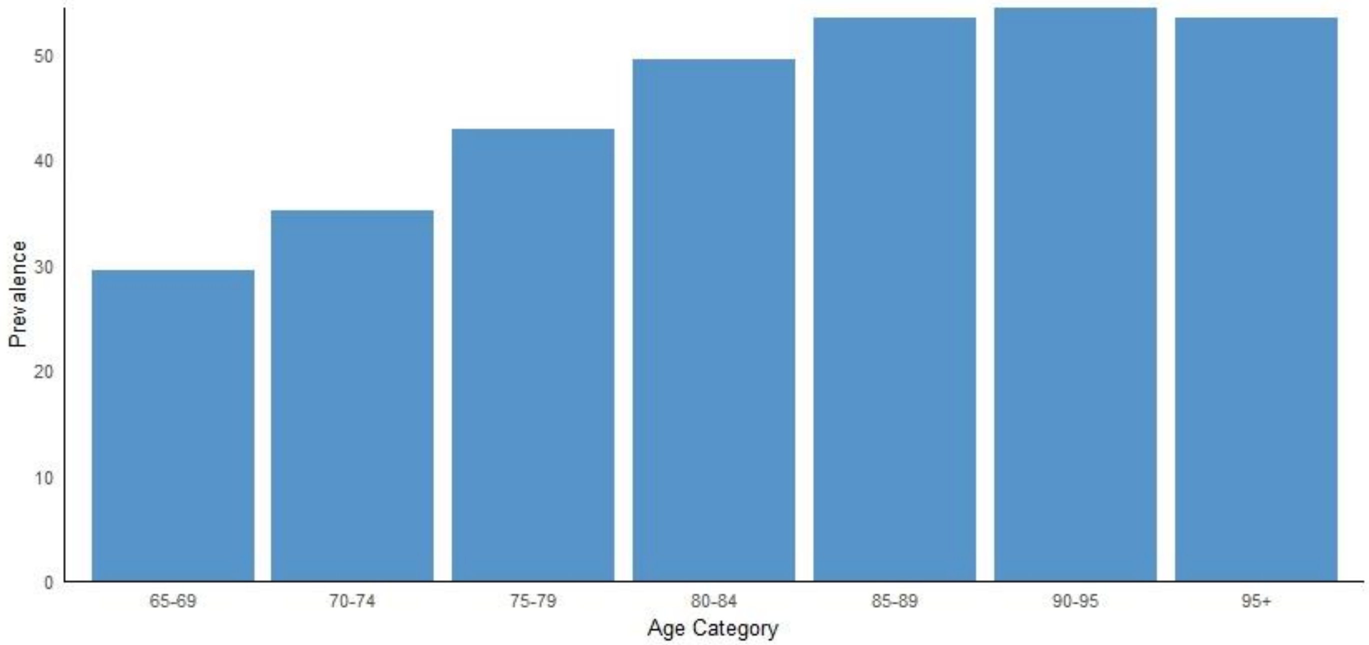

References

1. Izurieta HS, Chillarige Y, Kelman J, et al. Relative Effectiveness of Influenza Vaccines Among the United States Elderly, 2018-2019. *J Infect Dis*. Jun 29 2020;222(2):278-287. doi:10.1093/infdis/jiaa080
2. Izurieta HS, Lu M, Kelman J, et al. Comparative Effectiveness of Influenza Vaccines Among US Medicare Beneficiaries Ages 65 Years and Older During the 2019-2020 Season. *Clin Infect Dis*. Dec 6 2021;73(11):e4251-e4259. doi:10.1093/cid/ciaa1727
3. Zhang HT, McGrath LJ, Wyss R, Ellis AR, Sturmer T. Controlling confounding by frailty when estimating influenza vaccine effectiveness using predictors of dependency in activities of daily living. *Pharmacoepidemiol Drug Saf*. Dec 2017;26(12):1500-1506. doi:10.1002/pds.4298
4. Bath MF, Sidloff D, Saratzis A, Bown MJ, investigators UKAGS. Impact of abdominal aortic aneurysm screening on quality of life. *Br J Surg*. Feb 2018;105(3):203-208. doi:10.1002/bjs.10721
5. Howard DP, Banerjee A, Fairhead JF, Handa A, Silver LE, Rothwell PM. Age-specific incidence, risk factors and outcome of acute abdominal aortic aneurysms in a defined population. *Br J Surg*. Jul 2015;102(8):907-15. doi:10.1002/bjs.9838
6. UK Government. NHS Health Checks: applying All Our Health. Accessed 15/09/2022, 2023. https://www.gov.uk/government/publications/nhs-health-checks-applying-all-our-health/nhs-health-checks-applying-all-our-health
7. Melia J, Moss S. PSA testing in the UK. *Wiley Online*. 2009;14(1):9-13.
8. NHS England. Bone density (DEXA scan). Accessed 02/10/2023, 2023. https://www.nhs.uk/conditions/dexa-scan/
9. Wood ME, Chrysanthopoulou S, Nordeng HME, Lapane KL. The Impact of Nondifferential Exposure Misclassification on the Performance of Propensity Scores for Continuous and Binary Outcomes: A Simulation Study. *Med Care*. Aug 2018;56(8):e46-e53. doi:10.1097/MLR.0000000000000800
10. UK Government. Fingertips, Public Health Data, Abdominal Aortic Aneurysm Screening Coverage. Accessed 14/02/2023, 2023. https://fingertips.phe.org.uk/search/92317#page/4/gid/1/pat/159/par/K02000001/ati/15/are/E92000001/iid/92317/age/94/sex/1/cat/-1/ctp/-1/yrr/1/cid/4/tbm/1
11. UK Government. Breast screening coverage: aged 50 to 70 years old. Accessed 01/08/2023, 2023. https://fingertips.phe.org.uk/search/breast%20screening#page/4/gid/1/pat/159/par/K02000001/ati/15/are/E92000001/iid/91339/age/265/sex/4/cat/-1/ctp/-1/yrr/1/cid/4/tbm/1
12. UK Government. Cancer screening coverage: cervical cancer (aged 50 to 64 years old). Accessed 01/08/2023, 2023. https://fingertips.phe.org.uk/search/cervical#page/4/gid/1/pat/159/par/K02000001/ati/15/are/E92000001/iid/93561/age/273/sex/2/cat/-1/ctp/-1/yrr/1/cid/4/tbm/1
13. UK Government. Cancer screening coverage: bowel cancer. Accessed 01/08/2023, 2023. https://fingertips.phe.org.uk/search/screening#page/4/gid/1/pat/159/par/K02000001/ati/15/are/E92000001/iid/91720/age/280/sex/4/cat/-1/ctp/-1/yrr/1/cid/4/tbm/1
14. UK Government. Percentage of NHS Health Checks received by the total eligible population in the quarter. Accessed 01/08/2023, 2023. https://fingertips.phe.org.uk/profile/nhs-health-check-detailed/data#page/6/gid/1938132726/pat/159/par/K02000001/ati/15/are/E92000001/iid/91041/age/219/sex/4/cat/-1/ctp/-1/yrr/1/cid/4/tbm/1
15. UK Government. Population vaccination coverage: Flu (aged 65 and over). Accessed 31/07/2023, 2023. https://fingertips.phe.org.uk/search/92317#page/4/gid/1/pat/159/par/K02000001/ati/15/are/E92000001/iid/92317/age/94/sex/1/cat/-1/ctp/-1/yrr/1/cid/4/tbm/1
16. UK Government. Fingertips, Public Health Data, Population Vaccination Coverage, PPV. Accessed 14/02/2023, 2023.

https://fingertips.phe.org.uk/search/ppv#page/4/gid/1/pat/159/par/K02000001/ati/15/are/E92000001/iid/30313/age/27/sex/4/cat/-1/ctp/-1/yrr/1/cid/4/tbm/1
17. Young GJ, Harrison S, Turner EL, et al. Prostate-specific antigen (PSA) testing of men in UK general practice: a 10-year longitudinal cohort study. *BMJ Open*. Oct 30 2017;7(10):e017729. doi:10.1136/bmjopen-2017-017729
18. UK Government. Rate of dual-energy X-ray absorptiometry (DEXA) activity per weighted population by CCG. Accessed 01/08/2023, 2023. https://fingertips.phe.org.uk/documents/Atlas_2015_MuscSkel.pdf
19. NHS Digital. Appointments in General Practice report. Accessed 02/10/2023, 2023. https://digital.nhs.uk/data-and-information/data-tools-and-services/data-services/general-practice-data-hub/appointments-in-general-practice
20. NHS England. Evidence-Based Interventions: Consultation Document. Accessed 21/08/2023, 2023. https://www.england.nhs.uk/wp-content/uploads/2018/06/04-b-pb-04-07-2018-ebi-consultation-document.pdf
21. The Health Foundation. Trends in emergency admissions for ambulatory care sensitive conditions, 2001 to 2013. Accessed 24/07/2024, https://www.health.org.uk/publications/qualitywatch-focus-on-preventable-admissions
22. Shiue I. Cold homes are associated with poor biomarkers and less blood pressure check-up: English Longitudinal Study of Ageing, 2012-2013. *Environ Sci Pollut Res Int*. Apr 2016;23(7):7055-9. doi:10.1007/s11356-016-6235-y
