# Supplementary material for: Identifying markers of health-seeking behaviour and healthcare access in UK electronic health records
Source: BMJ Open. 2024 Sep 26;14(9):e081781. doi: 10.1136/bmjopen-2023-081781 (PMC11429345; doi:10.1136/bmjopen-2023-081781)
Supplement: Supplementary file 2 [file bmjopen-2023-081781.reviewer_comments.pdf]

PEER REVIEW HISTORY

BMJ Open publishes all reviews undertaken for accepted manuscripts. Reviewers are asked to complete a checklist review form and are provided with free text boxes to elaborate on their assessment. These free text comments are reproduced below.

ARTICLE DETAILS

**TITLE (PROVISIONAL)**	Identifying markers of health-seeking behaviour and healthcare access in UK electronic health records
**AUTHORS**	Graham, Sophie; Walker, Jemma; Andrews, Nick; Nitsch, Dorothea; Parker, Edward PK; McDonald, Helen

VERSION 1 - REVIEW

**REVIEWER NAME**	Bjerve Eide, Torunn
**REVIEWER AFFILIATION**	University of Oslo
**REVIEWER CONFLICT OF INTEREST**	None
**DATE REVIEW RETURNED**	20-Mar-2024

**GENERAL COMMENTS**	Thank you for the opportunity to review this article on the topic of identifying markers on health-care seeking behaviour and healthcare access in UK electronic health records.<br>Let me first state that after accepting this review, and receiving the full article, I realized that it is not quite within my field of knowledge, as the article focuses more on the methodology than the clinical implications. My review is therefore not very extensive, and I have not evaluated the methods used nor the justification of the conclusions based on the statistical analyses.<br>I have some brief comments:<br>The background section is well written and informative.<br>In the method section, some parts are difficult to follow for a reader that is not familiar with all the parts of the UK system. It could be a better solution to put the information on data sources in either a table or a text box, to alleviate the reading of the main text.<br>Regarding the marker selection: You indicate that “We developed candidate markers and formal criteria for marker selection, incorporating input from two clinical epidemiologists on UK clinical practice and data recording (DN and HIM). Candidate markers from the aforementioned US studies 6-8 were tested against these criteria to iteratively make improvements to the criteria and identify additional potential markers” . Could you be more specific? Which are the criteria that you tested the candidate markers against? I also suggest to move information from the supplementary material into the main article, to improve clarity regarding the process of developing the markers.<br>Further in the same section, you write “ If an individual has a visit to hospital for an ACS condition, then we can presume that they had a lack of healthcare access or health-seeking behaviour as they were unable to or did not access care when their symptoms were less severe”. I am not sure this is entirely correct. What about the system factors with increased problems with GP recruitment? With either inexperienced GPS, very busy GPs, or an increasing amount of private providers, it is possible that the threshold for referring to hospital treatment decreases and thus results in more hospital visits

	for ACS conditions.<br>In the section regarding marker operational definitions: In the first sentence, I suggest that you start by explaining how you define the terms code and code lists<br>You state that the "Faecal Immunochemical Tests are not used for symptomatic testing "– this is not true in the clinical setting I am used to, on the contrary it is the first choice to check for blood in the stools in patients where malignant or inflammatory disease is considered. Please explain.<br>I recommend that the text to a larger degree specifies which markers that address healthcare seeking behaviour and which address access.<br>In the results section, you indicate which markers show positive and negative correlation. In the discussion, I miss reflections regarding the significance of these correlations.<br>You found very high numbers of hospital visits for ACS conditions compared to what is found in previous literature. Could this signify that this is not a good marker? See also my previous comment regarding this marker.<br>The text as it stands is mainly written for UK readers. I would suggest to include some reflections in the discussion regarding to what extent the results are useful in other countries, where e.g. screening programs are different.

**REVIEWER NAME**	Mekete, Getachew
**REVIEWER AFFILIATION**	Debre Tabor University
**REVIEWER CONFLICT OF INTEREST**	The reviewer declares that there is no conflict of interest.
**DATE REVIEW RETURNED**	28-Mar-2024

**GENERAL COMMENTS**	Thanks so much for inviting me to review this article. Here are my thoughts on the article.<br>We would like to submit the commented article entitled "Identifying markers of health-seeking behavior and healthcare access in UK Electronic Health Records." to your journal.<br>First of all, thank you so much for involving me in reviewing this manuscript.<br>Positive feedback:<br>ϖ Complex but well-conducted and understandable statistical analysis with well-structured graphs, tables, images, and scientifically valid results.<br>ϖ The results are highly practical and offer great potential for enhancing user experience.<br>ϖ Clear and easily understood English language with easy reading.<br>ϖ Adequate and recent bibliography with many valid references.<br>ϖ Clear and understandable Strengths and limitations of this study<br>Of course, the article perhaps needs further enhancement in some areas, and the following are several suggestions for the authors to consider.<br>⎫ The specific study site is unclear and must be clarified.<br>⎫ The methods must include hypothesis/study site, location/study time-window (including date/month/year) primary and secondary outcome measures, and in- and exclusion criteria analysis of the data.<br>⎫ comparisons between different countries, and historical and human aspects have not been presented enough. The authors need to answer: the importance of Identifying markers of health-seeking

	behavior and healthcare access in UK electronic health records.<br><br>⎫ How did the author's team obtain the sample for the study? I hope the authors can answer the process of sample acquisition, screening criteria, problems in handling the sample and their solutions, and try to say that others, your sample is typical and representative.<br>Overall, the article is of good quality and is a logical, well-structured article<br>It is recommended to be published after minor revision.

VERSION 1 – AUTHOR RESPONSE

Reviewer: 1
Dr. Torunn Bjerve Eide, University of Oslo
Comments to the Author:
Thank you for the opportunity to review this article on the topic of identifying markers on health-care seeking behaviour and healthcare access in UK electronic health records.
Let me first state that after accepting this review, and receiving the full article, I realized that it is not quite within my field of knowledge, as the article focuses more on the methodology than the clinical implications. My review is therefore not very extensive, and I have not evaluated the methods used nor the justification of the conclusions based on the statistical analyses.
I have some brief comments:
The background section is well written and informative.
Response to reviewer: thank you very much for this feedback.
In the method section, some parts are difficult to follow for a reader that is not familiar with all the parts of the UK system. It could be a better solution to put the information on data sources in either a table or a text box, to alleviate the reading of the main text.
Response to reviewer: thank you for this feedback. As suggested we have put information on the data source in a table in the Methods section and removed corresponding text from the Methods. See "Table 1 Description of the CPRD Aurum-HES APC data sources".
Regarding the marker selection: You indicate that "We developed candidate markers and formal criteria for marker selection, incorporating input from two clinical epidemiologists on UK clinical practice and data recording (DN and HIM). Candidate markers from the aforementioned US studies 6-8 were tested against these criteria to iteratively make improvements to the criteria and identify additional potential markers" . Could you be more specific? Which are the criteria that you tested the candidate markers against? I also suggest to move information from the supplementary material into the main article, to improve clarity regarding the process of developing the markers.
Response to reviewer: thank you for this comment. The specific criteria are outlined in Table 2. We have modified the text to clarify this. As suggested, we have also moved information on some the excluded markers (and the reasons behind these exclusions) from the Supplementary material to the main text. The relevant updates are highlighted as follows "We selected candidate markers available within the CPRD Aurum-HES data using our own formal criteria for marker selection (see Table 2). The criteria were developed with input from two clinical epidemiologists on UK clinical practice and data recording (DN and HIM). Candidate markers from the aforementioned US studies6-8 were tested against our developed criteria to iteratively make improvements to the criteria and to identify additional potential markers. Candidate markers that failed to meet our pre-specified criteria included interactions strongly linked with underlying health need (e.g. for cardiovascular disease screening, depression screening, hospital visits) and programmes not available to all individuals (e.g. Shingrix vaccination; see Supplementary Table 1 for full list of excluded markers)."
Further in the same section, you write " If an individual has a visit to hospital for an ACS condition, then we can presume that they had a lack of healthcare access or health-seeking behaviour as they were unable to or did not access care when their symptoms were less severe". I am not sure this is

entirely correct. What about the system factors with increased problems with GP recruitment? With either inexperienced GPS, very busy GPs, or an increasing amount of private providers, it is possible that the threshold for referring to hospital treatment decreases and thus results in more hospital visits for ACS conditions.

Response to reviewer: thank you for highlighting contextual factors that would also contribute to this issue. We believe that the system factors highlighted fall within the health access issues that we aim to capture using ACS condition as a marker. To better capture these nuances, we have added the following text: "If an individual has a visit to hospital for an ACS condition, this could signify either barriers to healthcare access on the macro level (e.g., inundated GP practices), barriers on the individual level (e.g., language barriers) or a lack of health-seeking behaviour."

In the section regarding marker operational definitions: In the first sentence, I suggest that you start by explaining how you define the terms code and code lists

Response to reviewer: thank you for this. We have added the following definition for code lists: "sets of diagnostic or prescription codes that represent a given clinical concept"

You state that the "Faecal Immunochemical Tests are not used for symptomatic testing "– this is not true in the clinical setting I am used to, on the contrary it is the first choice to check for blood in the stools in patients where malignant or inflammatory disease is considered. Please explain.

Response to reviewer: FIT tests were introduced to the UK governmental routine screening program in 2019 (ref). FIT tests are also used routinely for investigation of symptoms in the UK at the present time, but we believe this was not usual practice at the time of the study index date (1 September 2019). The NICE guidance for use of FIT tests to detect cancer in symptomatic cases was published in the UK in 2022(ref). Since bowel cancer screening in the current study was only identified before 1 September 2019 (index date), we believe that majority of the tests captured would be for asymptomatic patients. We agree that studies that identify bowel cancer screening using FIT tests in later years or in other settings should be aware of this context. We have therefore modified the text in the Methods as so: "For example, for most screening markers we required the code to specify "screen" or "screening" but for bowel cancer we also allowed Faecal Immunochemical Tests (FIT) as these were in routine use in the national screening programme during the study period but were not a first choice for symptomatic testing at the time19."

We have also added the following limitation to the discussion: "Some markers might change in how they adhere to the developed criteria over time or in different settings. For example, in the UK NICE guidance has recommended FIT tests for symptomatic testing since 2022(ref)."

I recommend that the text to a larger degree specifies which markers that address healthcare seeking behaviour and which address access.

Response to reviewer: thank you for this comment. We believe that it is difficult to cleanly disentangle which of the markers represent healthcare access and which of those represent health-seeking behaviour. Selection of the markers for this study were based on a framework that was based on the updated Theory of Planned Behaviour Model by Schmid et al 2017(ref). Figure 1 in this paper shows the underlying determinants of receiving an influenza vaccination. There are many different determinants, and in light of this individual markers cannot be grouped into those that would solely reflect either healthcare access or health-seeking behaviour. We have added this sentence into the discussion: "Due to the number of different determinants that influence healthcare utilisation according to the updated TPB model, markers cannot solely be thought to solely represent either health-seeking behaviour or healthcare access."

In the results section, you indicate which markers show positive and negative correlation. In the discussion, I miss reflections regarding the significance of these correlations.

Response to reviewer: thank you for this comment. We have added the following text into the discussion: "The correlations between markers in the data are likely dictated by co-occurrence of these healthcare interactions (e.g., blood pressure measurements might be taken when influenza vaccinations are administered) or influenced by individual patient characteristics (e.g., age, underlying health conditions). Positive correlations (e.g., the high positive correlation between AAA screening and bowel cancer screening) is likely because the prevalence of both these markers are highest

amongst 65-69 year olds with declining prevalence with age (Figure 1). Negative correlations (e.g., the high negative correlation between NHS health checks and influenza vaccinations) are likely due to underlying health conditions. For example, NHS health checks are offered to those without a list of pre-existing conditions(ref), whereas the majority of the same conditions would qualify an individual for influenza vaccination prioritisation(ref). This aligns with expectations according to the updated TPB model(ref)."

You found very high numbers of hospital visits for ACS conditions compared to what is found in previous literature. Could this signify that this is not a good marker? See also my previous comment regarding this marker.

Response to reviewer: thank you for this comment. The estimate provided was for the entire English population, not just those aged ≥65 years, and limited to a subset of ACS admissions associated with chronic disease. We therefore expected our estimate of all admissions for ACS indications among older adults to be considerably higher.

We have reviewed the literature again and suggest that an estimate of ACS admissions 2001-2013 from the Health Foundation which is stratified by age will be more comparable to our study population of older adults, despite being less recent (ref). They estimate that the proportion of individuals with an unplanned hospitalisation for an ACS condition increases from 3% in those aged 65 years to 15% in those aged 85+ years. We have provided this updated estimate in Table 4 and have provided the updated reference in Supplementary Table 4.

The text as it stands is mainly written for UK readers. I would suggest to include some reflections in the discussion regarding to what extent the results are useful in other countries, where e.g. screening programs are different.

Response to reviewer: We have added the following text into the discussion: "Previous studies that have used EHR to identify markers of health-seeking behaviour and healthcare access in the US6-8 are in a considerably different context from the UK in terms of health services provided (e.g., screening programmes might differ), the healthcare system, claims-based recording systems and underlying determinants of health." We have also added the following: "Screening coverage differs significantly between countries, for example, a global repository for breast, cervical and colorectal screening programmes reported that breast cancer screening coverage ranged from 1.7% in Bangladesh to 85.5% in the UK. For cervical cancer coverage ranged from 2.1% in Côte d'Ivoire to 86.3% in Sweden and for colorectal cancer coverage ranged from 0.6% in Hungary to 64.5% in the Netherlands. Some countries may not even offer these preventative measures as free healthcare services or may offer then at different timepoints."

Reviewer: 2

Mr. Getachew Mekete, Debre Tabor University

Comments to the Author:

Dear Editor of BMJ Open

Thanks so much for inviting me to review this article. Here are my thoughts on the article.

We would like to submit the commented article entitled "Identifying markers of health-seeking behavior and healthcare access in UK Electronic Health Records." to your journal.

First of all, thank you so much for involving me in reviewing this manuscript.

Positive feedback:

ϖ Complex but well-conducted and understandable statistical analysis with well-structured graphs, tables, images, and scientifically valid results.

ϖ The results are highly practical and offer great potential for enhancing user experience.

ϖ Clear and easily understood English language with easy reading.

ϖ Adequate and recent bibliography with many valid references.

ϖ Clear and understandable Strengths and limitations of this study

Response to reviewer: we would like to thank the reviewer for this positive feedback.

Of course, the article perhaps needs further enhancement in some areas, and the following are several suggestions for the authors to consider.

⎫ The specific study site is unclear and must be clarified.
Response to reviewer: We would like to point the reviewer to relevant details regarding the study setting and population (e.g. number of general practices and population coverage for the CPRD Aurum database) under the first Methods subheading. As noted in our response to Reviewer 1, we have added additional details on the study database in "Table 1 Description of the CPRD Aurum-HES APC data sources".
⎫ The methods must include hypothesis/study site, location/study time-window (including date/month/year) primary and secondary outcome measures, and in- and exclusion criteria analysis of the data.
Response to reviewer: Our Methods section provides careful details regarding the timeframe, eligibility, and outcome definitions (for selected markers) used in our study. We have added information on the study design into the Section Data sources, study design and population. We have added the following text on the study design: "This was a cross sectional study design that included a Our study population included of individuals in England aged 66 years or older on the 1 September 2019 identified in the CPRD-HES data."
⎫ comparisons between different countries, and historical and human aspects have not been presented enough. The authors need to answer: the importance of Identifying markers of health-seeking behavior and healthcare access in UK electronic health records.
Response to reviewer: thank you very much for this comment. We have added the following into the discussion: "Screening coverage differs significantly between countries, for example, a global repository for breast, cervical and colorectal screening programmes reported that breast cancer screening coverage ranged from 1.7% in Bangladesh to 85.5% in the UK. For cervical cancer this range from 2.1% in Côte d'Ivoire to 86.3% in Sweden and for colorectal cancer this ranged from 0.6% in Hungary to 64.5% in the Netherlands. Some countries might not even offer these preventative services as a free healthcare service and these healthcare services would have become available at different timepoints within different countries."
⎫ How did the author's team obtain the sample for the study? I hope the authors can answer the process of sample acquisition, screening criteria, problems in handling the sample and their solutions, and try to say that others, your sample is typical and representative.
Response to reviewer: thank you for this. The newly added Table 1 contains details on the data source and representativeness of CPRD Aurum. Additional details on the study population are provided as follows: "This was a cross sectional study design that included a study population of individuals in England aged 66 years or older on the 1 September 2019 identified in the CPRD-HES data."
Overall, the article is of good quality and is a logical, well-structured article
It is recommended to be published after minor revision.

VERSION 2 – REVIEW

**REVIEWER NAME**	Bjerve Eide, Torunn
**REVIEWER AFFILIATION**	University of Oslo
**REVIEWER CONFLICT OF INTEREST**	None
**DATE REVIEW RETURNED**	07-Aug-2024

**GENERAL COMMENTS**	Thank you for this new version of your article. All my concerns have been sufficiently handled.<br>To the editor: I recommend that the article is also evaluated by a reviewer with thorough knowledge about the NHS , and with a

	sufficient methodologic background to evaluate the performed analyses.

VERSION 2 – AUTHOR RESPONSE
